# TAF2, within the TFIID complex, regulates the expression of a subset of protein-coding genes

**DOI:** 10.1038/s41420-024-02017-z

**Published:** 2024-05-21

**Authors:** I-Hsin Cheng, Wen-Chieh Pi, Chung-Hao Hsu, Yiran Guo, Jun-Lin Lai, Gang G. Wang, Bon-chu Chung, Robert G. Roeder, Wei-Yi Chen

**Affiliations:** 1 Institute of Biochemistry and Molecular Biology, National Yang Ming Chiao Tung University, Taipei, Taiwan; 2 School of Medicine, College of Medicine, National Yang Ming Chiao Tung University, Taipei, Taiwan; 3 Department of Pharmacology and Cancer Biology, Duke University School of Medicine, Durham, NC 27710 USA; 4 Duke Cancer Institute, Durham, NC 27710 USA; 5 Program in Molecular Medicine, National Yang Ming Chiao Tung University and Academia Sinica, Taipei, Taiwan; 6 Insitute of Molecular Biology, Academia Sinica, Taipei, Taiwan; 7 Graduate Institute of Biomedical Sciences, Neuroscience and Brain Disease Center, China Medical University, Taichung, Taiwan; 8 National Laboratory Animal Center, National Applied Research Laboratories, Taipei, Taiwan; 9 Laboratory of Biochemistry and Molecular Biology, The Rockefeller University, New York, NY 10065 USA; 10 Cancer and Immunology Research Center, National Yang Ming Chiao Tung University, Taipei, Taiwan

**Keywords:** Transcriptional regulatory elements, DNA-binding proteins, Transcriptomics

## Abstract

TFIID, one of the general transcription factor (GTF), regulates transcriptional initiation of protein-coding genes through direct binding to promoter elements and subsequent recruitment of other GTFs and RNA polymerase II. Although generally required for most protein-coding genes, accumulated studies have also demonstrated promoter-specific functions for several TFIID subunits in gene activation. Here, we report that TBP-associated factor 2 (TAF2) specifically regulates TFIID binding to a small subset of protein-coding genes and is essential for cell growth of multiple cancer lines. Co-immunoprecipitation assays revealed that TAF2 may be sub-stoichiometrically associated with the TFIID complex, thus indicating a minor fraction of TAF2-containing TFIID in cells. Consistently, integrated genome-wide profiles show that TAF2 binds to and regulates only a small subset of protein-coding genes. Furthermore, through the use of an inducible TAF2 degradation system, our results reveal a reduction of TBP/TFIID binding to several ribosomal genes upon selective ablation of TAF2. In addition, depletion of TAF2, as well as the TAF2-regulated ribosomal protein genes RPL30 and RPL39, decreases ribosome assembly and global protein translation. Collectively, this study suggests that TAF2 within the TFIID complex is of functional importance for TBP/TFIID binding to and expression of a small subset of protein-coding genes, thus establishing a previously unappreciated promoter-selective function for TAF2.

## Introduction

Transcription of protein-coding genes is essential to various cellular processes and is tightly regulated by highly complex multicomponent machineries [1]. These machineries include RNA Polymerase II (Pol II), the Mediator complex, and general transcription factors (GTFs; such as TFIIA, TFIIB, TFIID, TFIIE, TFIIF, and TFIIH) that are required for activating most protein-coding genes [2, 3]. Among these GTFs, TFIID is a multisubunit protein complex, composed of the TATA box-binding protein (TBP) and 13-14 TBP-associating factors (TAFs). TFIID recognizes and binds directly to the core promoter elements, including the TATA box, Initiator (Inr), and/or downstream core promoter element (DPE), through specific subunits and further recruits other GTFs and Pol II to form the transcriptional preinitiation complex (PIC) [3, 4]. Despite their general function in gene activation, several TAF subunits within the TFIID complex have been reported to function in a promoter-specific manner. For example, TAF10 is required for GATA1-regulated expression of genes involved in erythropoiesis [5]; TAF4 functions as a co-activator for E protein-mediated gene activation and for HNF4A-regulated genes involved in postnatal differentiation of hepatocytes [6, 7]; and TAF12 is essential for the expression of MYB target genes in leukemogenesis [8]. Although all TAFs are essential for the viability of yeast, gene expression analyses revealed that a variable number of genes, ranging from 3% to 60%, are regulated by individual TAFs [9]. These observations strongly suggest factor- and/or gene-specific functions for TAFs within the TFIID complex.

TAF2, of interest here, is the second largest subunit of TFIID and is evolutionally conserved from yeast to human [10]. Previous studies identified TAF2 as the first individual TAF to recognize and interact with the core promoter elements [11]. Furthermore, TAF2 was also reported to stabilize TFIID on Inr-containing promoters [11–16], although the mechanism by which TAF2 recognizes the Inr element is still controversial. Regarding the biological functions of TAF2, inactivation of *Taf2* (*TSM1*) in yeast through a temperature-sensitive mutation resulted in a dramatic defect in cell growth and downregulation of only 3% of protein-coding genes based on microarray analyses [9]. Furthermore, depletion of the murine *Taf2* by shRNA treatment efficiently suppressed the proliferation of normal and transformed myeloid cells [8], suggesting a TAF2 dependency not only for yeast but also for mammalian cells. In addition, homologous mutations of *TAF2* have been identified in, and proposed to be correlated with, patients with family microcephaly [17–19]. These observations suggest that TAF2 is essential for cell growth by regulating a relatively small set of genes. However, the underlying mechanism for TAF2 dependency and its direct target genes remain largely unknown.

Here, we report that TAF2 may exist in only a small fraction of TFIID complexes and is essential for the cell growth of multiple cancer cell lines. Genome-wide profiling revealed that TAF2 specifically localizes to the promoters of a small subset of genes, such as those encoding L and S ribosomal proteins (*RPLs* and *RPSs*), involved in the ribosome assembly pathways. Complementary cell-based assays with degradation-inducible TAF2 in HCT116 cells confirm its dependency on TBP/TFIID binding to ribosomal protein (*RP*) genes. Unexpectedly, genome-wide gene expression profiles suggest a TAF2 dependency only for the expression of a small subset of genes, including *RPL30* and *RPL39*. Polysome profiling assays further confirmed a requirement for TAF2, as well as TAF2-regulated RPL30 and RPL39, function in regulating the ribosome assembly and protein synthesis pathways. Altogether, these studies uncover a previously unappreciated gene-specific role for TAF2, within the TFIID complex, in regulating TBP/TFIID binding to promoters of genes involving ribosome assembly and function.

## Results

### TAF2 subunit is sub-stoichiometrically present in the TFIID complex

**Fig. 1:**
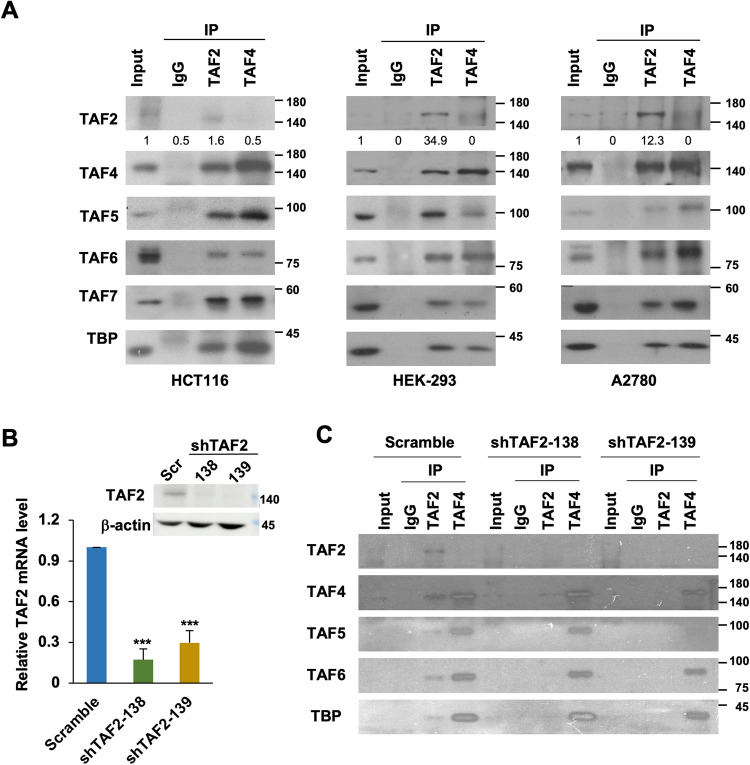
TAF2 subunit is presented in a small fraction of TFIID complex. **A** Immunoblots of the indicated TFIID subunits in the immunoprecipitates (IP) using either normal rabbit IgG, anti-TAF2, or anti-TAF4 antibodies in the nuclear extracts of colorectal cancer HCT116, human embryonic kidney HEK-293, and ovarian cancer A2780 lines. Numbers under TAF2 immunoblot indicate a relative level of input after normalization to the TAF5 level. **B** RT-qPCR assays showing the mRNA level of *TAF2* in HCT116 cells treated with Scramble or TAF2-targeting (shTAF2-138 or shTAF2-139) shRNAs for 144 h. Results are relative to Scramble and represented as mean ± SD of 3 independent experiments. Student’s *t* test, ***, *P* < 0.001. The i*nset* shows the immunoblot of TAF2 in the indicated shRNA-treated HCT116 cells. The β-actin is a loading control. **C** Immunoblots of the indicated TFIID subunits in the anti-TAF2 and anti-TAF4 immunoprecipitates using the lysates from HCT116 cells treated with Scramble or TAF2-targeting (shTAF2-138 and TAF2-139) shRNAs for 144 h.

Although TAF2 was reported to be an integrated subunit of the TFIID complex, some previous studies also argued that it is usually absent in the holo-TFIID complex isolated from eukaryotic cells. For example, Martin et al. reported that TAF2 was not co-eluted with TAF1 (TAFII250) and TAF4 (TAFII130/135) in a gel filtration assay of HeLa nuclear extract [20], indicating that the majority of TAF2 might not be tightly associated within the TFIID complex. These observations may also suggest that TFIID integrity is independent of TAF2. Given that the commercially available TAF2 antibodies are not suitable for immunoprecipitation (IP) and ChIP assays (data not shown), we raised a polyclonal antibody against the C-terminus of human TAF2 which is efficient and specific in immunoassays (see Materials and Methods and Supplemental Fig. S1). We carried out co-IP assays to examine whether TAF2 associates with the TFIID complex in three independent cancer cell lines. As shown in Fig. 1A, our anti-TAF2 antibody was able to immunoprecipitate endogenous TAF2, as well as core subunits of the TFIID complex, including TBP, TAF4, TAF5, TAF6, and TAF7, in nuclear extracts from colorectal HCT116, embryonic kidney HEK-293, and ovarian A2780 cancer lines. Notably, and in contrast, TAF2 was not, or was barely, detected in anti-TAF4 immunoprecipitates, while other TFIID core subunits were enriched (Fig. 1A). A recent study showed that several TFIID core subunits, including TAF4, TAF5, and TAF6, can form multisubunit TFIID building blocks in the cytoplasm of HeLa cells [21]. To exclude the possibility that our TAF2-less anti-TAF4 immunoprecipitates were contaminated with cytoplasmic TFIID building blocks [21], we examined the anti-TAF4 immunoprecipitates in the cytosolic and nuclear extracts from HEK-293 and HCT116 cells. Our results showed that nuclear, but not cytoplasmic, TAF4 can associate with TAF6 and TBP in our preparations (Supplemental Fig. S2). To further validate the specificity of our anti-TAF2 antibody and to test whether TAF2 is essential for the integrity of the TFIID complex, we identified two individually designed shRNA clones (shTAF2-138 and shTAF2-139) that efficiently decrease the TAF2 mRNA and protein levels in HCT116 cells (Fig. 1B). Whereas comparable levels of TFIID core subunits could still be detected in the anti-TAF4 immunoprecipitates in both control and shTAF2-treated cells, no TAFs or TBP could be detected in the anti-TAF2 immunoprecipitates in the nuclear extract of HCT116 cells treated with shTAF2 (Fig. 1C). These observations suggest that TAF2 is weakly or sub-stoichiometrically associated with the TFIID complex. However, further quantitative and stoichiometric analyses will be required to validate the TAF2-less TFIID complex.

### TAF2 is required for the cell growth and survival of multiple cancer lines

**Fig. 2:**
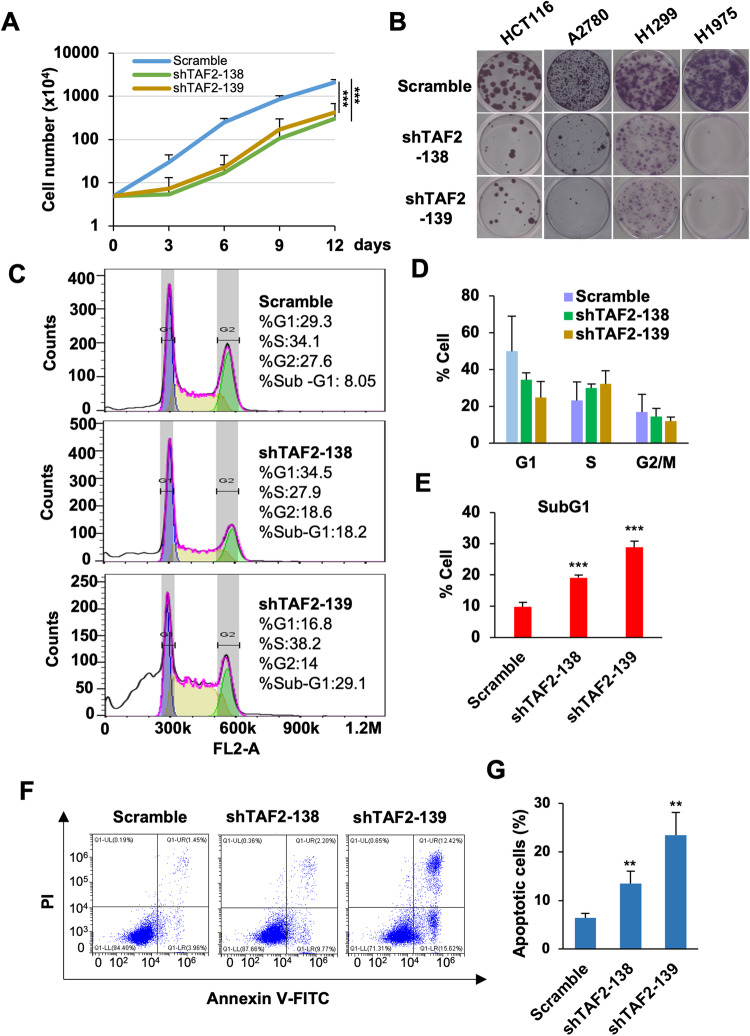
TAF2 is required for cell growth of multiple tested cancer lines. **A** Proliferation of HCT116 cells treated with Scramble or TAF2-targeting (shTAF2-138, shTAF2-139) shRNAs. Mean ± SD of 3 independent experiments. ANOVA, ***, *P* < 0.001; **, *P* < 0.01. **B** Colony-forming assays of HCT116, A2780, H1299, and H1975 cells treated with the indicated shRNAs. Images of crystal violate staining were scanned on day 14 culture. **C** Representative cell cycle profiles of HCT116 cells treated with Scramble or TAF2-targeting (shTAF2-138 and shTAF2-139) shRNAs. The cell cycle stages were determined by the Watson Pragmetic algorithm in FlowJo software. **D**, **E** Histograms showing the percentage of cells in cycling (**D**) and sub-G1 (**E**) stages. Means ± SD of 3 independent experiments. Student’s *t* test, ***P < 0.001. **F** Representative apoptosis assay of HCT116 cells treated with Scramble or TAF2-targeting (shTAF2-138 and shTAF2-139) shRNAs. Annexin-V-FITC and propidium iodide (PI) signals were determined by flow cytometry. **G** Summary of apoptotic HCT116 cells treated with the indicated shRNAs. Mean ± SD of 3 independent experiments. Student’s *t* test, ***P < 0.001; **P < 0.01. (See also Supplemental Fig. S2 for apoptosis assay in other cancer lines).

To investigate whether TAF2 is required for cell growth, we carried out cell proliferation assays and cell cycle analyses of TAF2-depleted HCT116 cells. Compared to control shScramble-treated cells, shTAF2-treated (138 and 139) cells exhibited reduced proliferation (Fig. 2A). In addition, a prolonged proliferation assay based on colony formation also showed that shTAF2 treatments impeded the growth of all tested cancer cells that included HCT116, H1299, H1975, and A2780 lines (Fig. 2B). To assess the cellular defects, cell cycle profiles and apoptosis of control-treated or TAF2-depleted cells were further analyzed. While the distribution of the cycling cells was not obviously altered (Fig. 2C, D), the numbers of SubG1 (Fig. 2C, E) and apoptotic cells were increased significantly by the shTAF2 treatments (Fig. 2F, G and Supplemental Fig. S3). Collectively, these results suggested that TAF2 is indispensable for cell survival of multiple cancer cell lines.

### TAF2 binds to a small subset of Pol II-regulated promoters

**Fig. 3:**
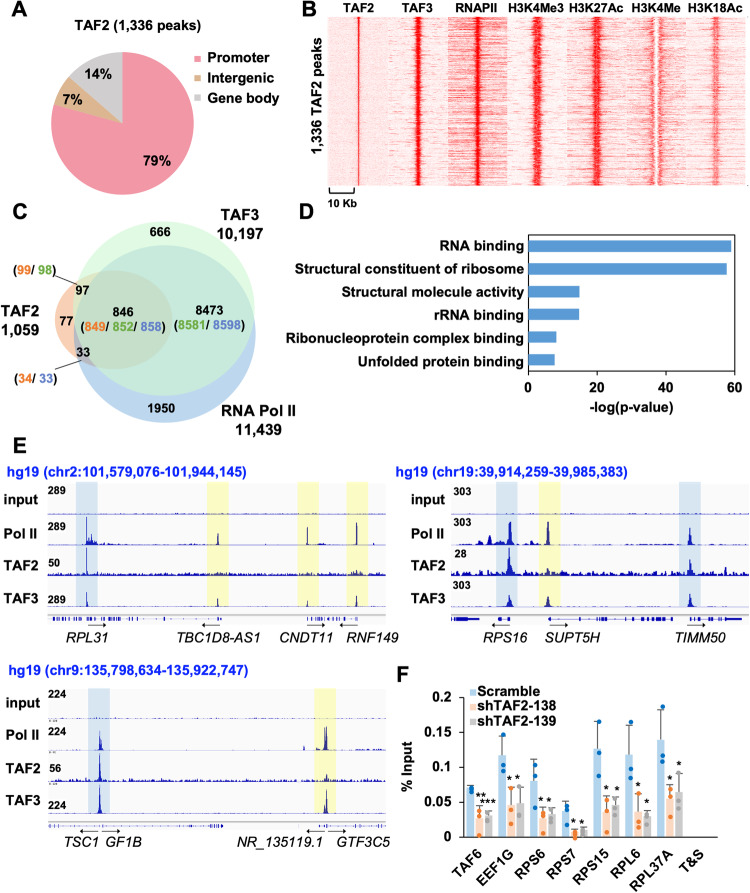
TAF2 binds to a small subset of TFIID and Pol II-occupied promoters. **A** Pie chart showing the distribution of TAF2 ChIP-seq peaks located at promoter (TSS ± 500 bp), gene body (from 500 bp downstream of TSS to TES), and intergenic region. **B** Heatmap showing the ChIP-seq signals of TAF2, TAF3 (GEO: GSE43542), RNA Pol II (GEO: GSE43542), H3K4Me2, H3K27Ac, H3K4Me, and H3K18Ac centered at identified TAF2 ChIP-seq peaks. **C** Venn diagrams showing the overlaps of promoter-bound ChIP-peaks of TAF2 (orange), TAF3 (green), and RNA Pol II (blue). **D** Genomic Regions Enrichment of Annotations Tool (GREAT) analysis of the identified TAF2 ChIP-seq peaks. **E** ChIP-seq profiles of RNA PoI II, TAF2, and TAF3 at the indicated genomic regions in HCT116 cells. Blue-colored boxes denote the colocalization of TAF2, TAF3, and Pol II ChIP-seq peaks; yellow-colored boxes denote the lack of TAF2 ChIP-seq signals at genes co-bound by TAF3 and Pol II. **F** ChIP-qPCR assays confirming the TAF2 occupancy at promoters of the indicated genes. TAF2 ChIP assays were performed in HCT116 cells treated with indicated shRNAs. T&S is an intergenic region control.

Our above-described biochemical analyses may have disrupted fragile interactions between TAF2 and other subunits within the TFIID complex, or TAF2 might be missing or sub-stoichiometric in the isolated TFIID complex(es). To better examine whether TAF2 can function as an integrated subunit of the TFIID complex, we next profiled the genomic binding of TAF2 using ChIP-seq assays, which employ formaldehyde to preserve in-situ interactions between chromatin and DNA-associated proteins. In agreement with our Co-IP assays, ChIP-seq with anti-TAF2 antibody in HCT116 cells identified a total of 1336 peaks, in which we set a *p* value cutoff at 10e-9 after visualizing the peaks identified by the MACS program. Consistent with the TFIID function in promoter recognition, 79% of TAF2 ChIP-seq peaks were found at gene promoter regions (TSS ± 500 bp) (Fig. 3A and Supplemental Dataset). In addition, the identified TAF2-bound 1336 peaks were also highly conserved in other tested cancer lines, such as ovarian A2780 and lung H1299 cancer cell lines (Supplemental Fig. S4). Further analyses revealed that TAF2-bound regions are also co-occupied by TAF3, Pol II, and active promoter marks that include H3K4Me3, H3K27Ac, and H3K18Ac, but not the active enhancer-specific mark H3K4Me1 (Fig. 3B). These observations suggest that TAF2 selectively binds to active promoter regions. Notably, an analysis of overlapping TAF3- and Poll II-bound promoters revealed that TAF2 occupies only a relatively small subset of TFIID- and Pol II-bound promoters (Fig. 3C, Supplemental Fig. S5, and Supplemental Dataset). In addition, positive correlations are also noted in the gene transcript level relative to the ChIP-seq signals of TAF2, TAF3, and Pol II in HCT116 cells (Supplemental Fig. S5). GREAT (genomic regions enrichment of annotations tools) analyses indicated that TAF2-bound regions are significantly associated with genes involved in RNA binding and structural constituents of ribosomes (Fig. 3D). The small number of TAF2 ChIP-seq peaks is not due to any inefficiency of our custom-made anti-TAF2 antibody. As exemplified in Fig. 3E, obvious TAF2 ChIP-seq peaks were found at the promoters of *RPL31* and *RPS16*, *TIMM50 and TSC1*, but not at the nearby gene loci for *TBC1D8, CNDT11, RNF149, SUPT5H, GTF3C5*. Importantly, comparable ChIP-seq signals of RNA Pol II and TAF3 further indicate that all the examined genes are transcriptionally active. However, there is little possibility that our formaldehyde-based ChIP-seq assays may not be able to capture all promoter-bound TFIID in different conformational states [15]. Furthermore, ChIP-qPCR assays in TAF2-depleted HCT116 cells further confirmed the TAF2 occupancies at promoter regions of genes encoding ribosome constituents that include *RPS6, RPS7*, *RPS15*, *RPL37A*, as well as non-ribosomal *TAF6* and *EEF1G* (Fig. 3F). Collectively, our analyses have delineated a selective binding of TAF2 to genes encoding both L and S ribosomal proteins.

### TAF2 depletion decreases TBP/TFIID binding to a small subset of genes, notably ribosomal protein-coding genes

**Fig. 4:**
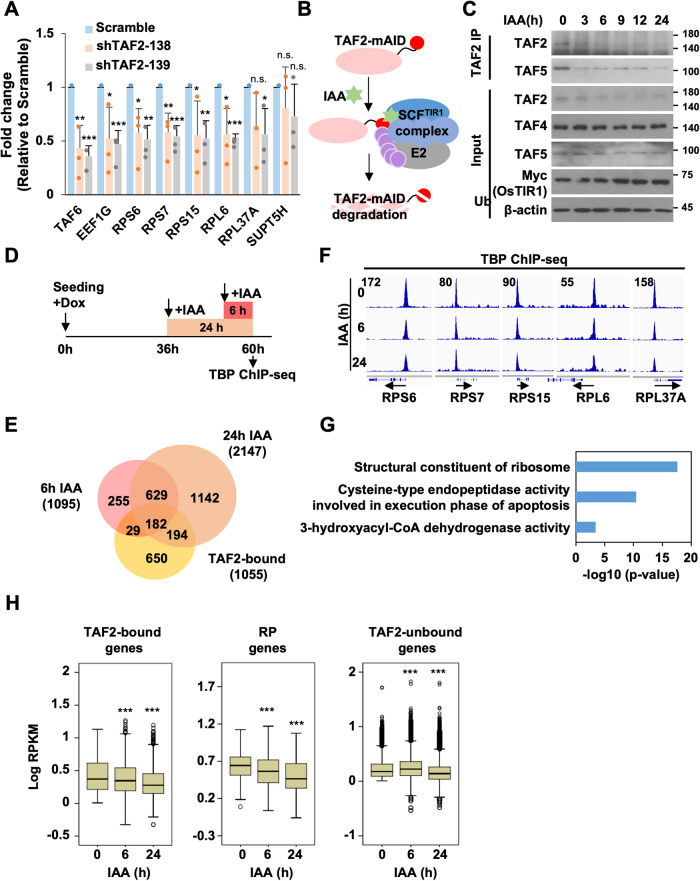
TAF2 regulates TBP/TFIID binding to a small subset of Pol II-controlled genes. **A** ChIP-qPCR of TBP (representative of TFIID) binding to indicated genes in HCT116 cells treated with Scramble or TAF2-targeting shRNAs. Results are relative to Scramble and shown as mean ± SD of 3 independent experiments. Student’s *t* test, ***, *P* < 0.001; **, *P* < 0.01; *, *P* < 0.05; n.s., not significant. **B** Schematic for Auxin-inducible degradation TAF2 system in HCT116 cells. mAID, mini-Auxin-inducible degron tag; IAA, Indole-3-acetic acid. **C** Immunoprecipitation assay showing the mAID-mediated ablation of TAF2 in a time-dependent manner in HCT116 cells treated with IAA. IPs were performed with anti-TAF2 antibody. TFIID subunits and OSTIR1-3Myc in the cell lysates were detected by immunoblotting with indicated antibodies. β-actin is a loading control. Note: TAF2 ablation does not affect the expression levels of TAF4 and TAF5 in input. **D** Schematic representation of the protocol used to access the effect of TAF2 ablation on TBP/TFIID binding. Cells were seeded and incubated with doxycycline (Dox) to induce the expression of OsTIR1 for 36 hr. TAF2 ablation was induced by IAA treatment for indicated times and followed by TBP ChIP-seq assay. **E** Venn diagram showing the overlap between TBP peaks with reduced ChIP-seq signal in 6 h and 24 h IAA treatment. The threshold of reduced TBP ChIP-seq signal is set as fold-change < 0.75, relative to control cells. **F** ChIP-seq profiles of TBP ChIP-seq signal at indicated ribosomal protein genes in HCT116 cells treated with IAA for 0, 6, and 24 hr. Black arrows indicate the direction of transcription. **G** GREAT analysis of overlapped TBP peaks in **E**. **H** Box plots of TBP ChIP-seq signal at TAF2-bound (left), ribosomal protein (RP, middle), and TAF2-unbound (right) gene promoters (TSS ± 200 bp) in AID-TAF2 HCT116 cells treated with IAA for 0, 6, and 24 hr. Statistical significance was determined by the Mann–Whitney *U* test. Note: The TBP ChIP-seq signals of TAF4-unbound genes were significantly increased at 6 hr IAA treatment.

To determine whether TAF2 is required for TFIID function, we first assessed the binding of TBP (representative of TFIID) to identify TAF2-bound promoters by ChIP-qPCR assays in HCT116 cells treated with control or TAF2-targeting shRNAs. Notably, we observed that TAF2 depletion significantly reduced TBP binding to TAF2-bound genes, including *RPS6, RPS7, RPS15, RPL6, RPL37A, TAF6*, and *EEF1G*, but not the TAF2-unbound *SUPT5H* (Fig. 4A). These results indicate that TAF2 regulates TBP/TFIID binding in a gene-specific manner. To further investigate such an effect on a genome-wide scale and to rule out indirect effects on TBP/TFIID binding during the prolonged incubation needed for shRNA effectiveness, we established an auxin-inducible degradation (AID) TAF2 system in HCT116 cells (Fig. 4B). In this system, a stable AID-TAF2 HCT116 line was established by utilizing CRISPR/Cas9-mediated gene editing to knock-in the mini-auxin-inducible degron (mAID) in-frame to exon 26, encoding the C-terminus, of TAF2, and then transduced the cells with lentiviruses expressing OsTIR1 in a doxycycline-inducible manner (Supplemental Fig. S6). Co-IP assays revealed intact interactions of TAF2-mAID with other TAFs and TBP (Fig. 4C and Supplemental Fig. S5), indicating that the mAID tag on TAF2 does not affect its integration into the TFIID complex. In addition, induction of TAF2 ablation also significantly repressed the proliferation of AID-TAF2 HCT116 cells (Supplemental Fig. S7). As expected, TAF2 was ablated in a time-dependent manner after auxin (IAA) treatment (Fig. 4C, top panel), whereas the protein levels of TAF4 and TAF5 were not affected during the treatment (Fig. 4C, bottom panel). To assess the TAF2 function for TBP/TFIID binding at the genome-wide scale, we carried out TBP ChIP-seq assays in cells pretreated with doxycycline to induce the expression of OsTIR1, followed by IAA treatment for 0, 6, or 24 h (Fig. 4D). Compared to cells treated without IAA, there were 1095 and 2147 genes with reduced TBP binding in cells treated with IAA for 6 and 24 h, respectively (Fig. 4E and Supplemental Dataset). Although only ~38% of TAF2-bound genes exhibited a reduction of TBP binding, this may reflect the incomplete TAF2 degradation by the IAA treatment. In contrast, 2214 and 836 genes were identified with increased TBP binding in cells treated with IAA for 6 and 24 h, respectively (Supplemental Fig. S8). As anticipated, only a small portion of these genes were identified as TAF2-bound. Notably, the GREAT analysis exhibited a consistently decreased TBP binding after 6-h and 24-h IAA treatments further revealed significant enrichment for genes related to ribosome constituents (Fig. 4F, G). Moreover, we observed that IAA treatment induced a time-dependent reduction of TBP/TFIID binding to TAF2-bound genes, especially those encoding RPs (Fig. 4H, left and middle panels; Supplemental Dataset). Curiously, the TBP ChIP-seq signal of many TAF2-bound promoters was unchanged after IAA treatment; it probably indicates that the remaining TAF2 proteins, resulting from incomplete AID-mediated depletion, could be sufficient for these promoters (Supplemental Dataset). Interestingly, at TAF2-unbound genes, we first observed a significantly enhanced TBP/TFIID binding after 6-h IAA treatment and then detected a significant decrease after 24-h treatment (Fig. 4H, right panel; Supplemental Dataset). This indicates that rapid TAF2 ablation redirects TBP/TFIID binding as an immediate effect and that, as expected, a prolonged TAF2 loss may cause indirect effects such as impaired protein translation since RPs are essential for ribosome functions. Altogether, our results suggest that TAF2 directs TBP/TFIID binding to a small subset of Pol II-regulated genes enriched in the ribosome pathway.

### Transcriptomic analysis reveals a regulatory role for TAF2 in the expression of a small subset of Pol II-regulated genes

**Fig. 5:**
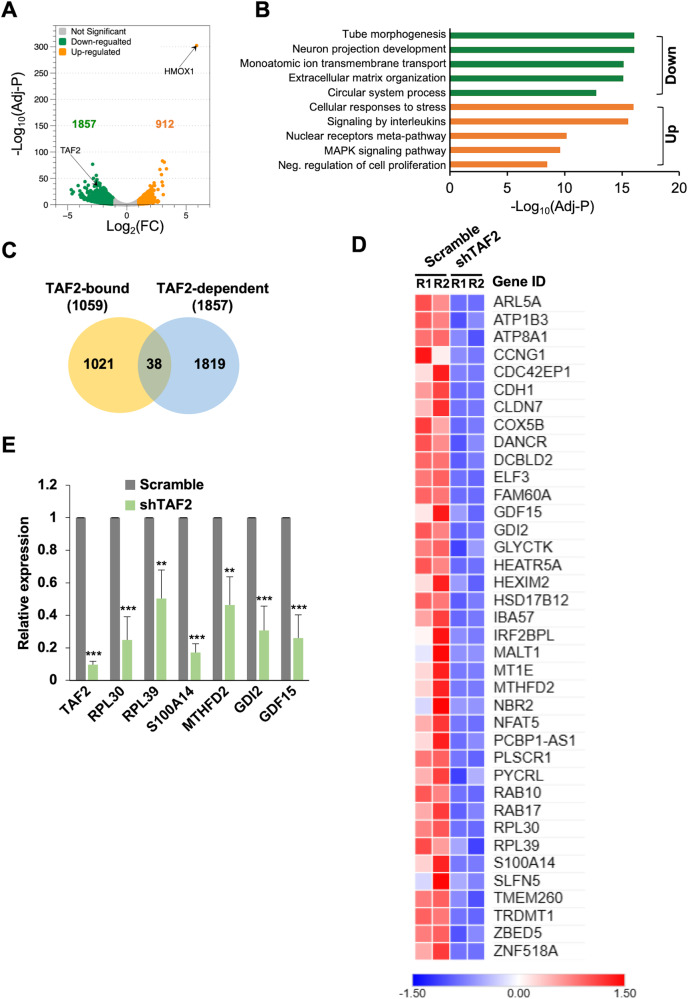
TAF2 regulates a small subset of Pol II-transcribed genes. **A** Volcano plot showing the downregulated (green, 1857) and upregulated (orange, 912) genes in shTAF2-treated HCT116 cells, relative to Scramble. Total RNAs were isolated from cells treated with indicated shRNAs for 144 h and submitted to RNA-seq analysis. Differentially expressed genes are defined as Log_2_ fold-change (FC) ≥ 1 and −Log_10_ (Adj *P*) ≥ 10. **B** Gene ontology of differentially expressed genes in TAF2-depleted HCT116 cells. **C** Venn diagram showing the overlap of genes between TAF2-bound (identified by ChIP-seq) and TAF2-dependent (downregulated in TAF2-depleted cells identified by RNA-seq). **D** Heatmap of RNA-seq results of overlapped genes in **C**. Two biological experiments (R1 and R2) are shown. **E** RT-qPCR assays validating the downregulation of TAF2 target genes in shTAF2-treated HCT116 cells, relative to Scramble. Mean ± SD of three independent experiments. Student’s *t* test, ***, *P* < 0.001; **, *P* < 0.01.

A previous study in yeast revealed that the inactivation of Taf2 reduced the expression of the least number of genes when compared to the inactivation of other *Tafs* [9]. In addition, a more recent study showed a dramatic decrease in genome-wide Pol II recruitment after the depletion of several yeast *Tafs*, whereas a milder effect was observed in *Taf2* depletion [22]. In agreement, our ChIP-seq data also indicated that only a small subset of Pol II-regulated genes is bound by TAF2 in human cells. To further identify TAF2-regulated genes, transcriptomic analyses were performed in HCT116 cells treated with scramble or TAF2-targeting shRNAs. In two biological RNA-seq experiments, our results showed that 1857 genes were downregulated and 912 upregulated (|log_2_ fold-change| ≥ 1, Adj *P* < 0.05) (Fig. 5A and Supplemental Dataset). We found that the cellular oxidative stress marker gene *HMOX1*, heme oxygenase-1, is the most upregulated gene, indicating that TAF2-depleted cells may undergo great cellular stress. Indeed, the Gene Ontology (GO) analysis identified the pathway involved in “the cellular response to stress” is upregulated (Fig. 5B). Except for the upregulated “negative regulation of cell proliferation” pathway, we did not identify other pathways that are related to the reduced proliferation of TAF2-depleted cells. To further determine the TAF2-dependent direct targets, we overlapped the TAF2-bound promoters and genes downregulated in TAF2-depleted cells. This analysis identified only 38 genes (Fig. 5C). Unexpectedly, among the TAF2-bound RP genes, only *RPL30* and *RPL39* were significantly downregulated in shTAF2-treated cells (Fig. 5D and Supplemental Dataset). These milder effects might reflect incomplete gene silencing by shRNA treatment (Fig. 1B), such that the remaining TAF2 might be sufficient for TAF2-bound promoters. Toward biological assays, we selected six of the TAF2-dependent direct target genes, namely *RPL30*, *RPL39* [23], *MTHFD2* [24], *S100A14* [25], *GDI2* [26], and *GDF15* [27], which were previously reported to be involved in the regulation of cell growth. Downregulation of these six genes was also validated by RT-qPCR assays in HCT116 cells treated with shTAF2 (Fig. 5E). Collectively, our observations suggest that a TAF2 dependency for only a small number of Pol II-regulated genes.

### TAF2-regulated *RPL30* and *RPL39* are required for functional ribosomes

**Fig. 6:**
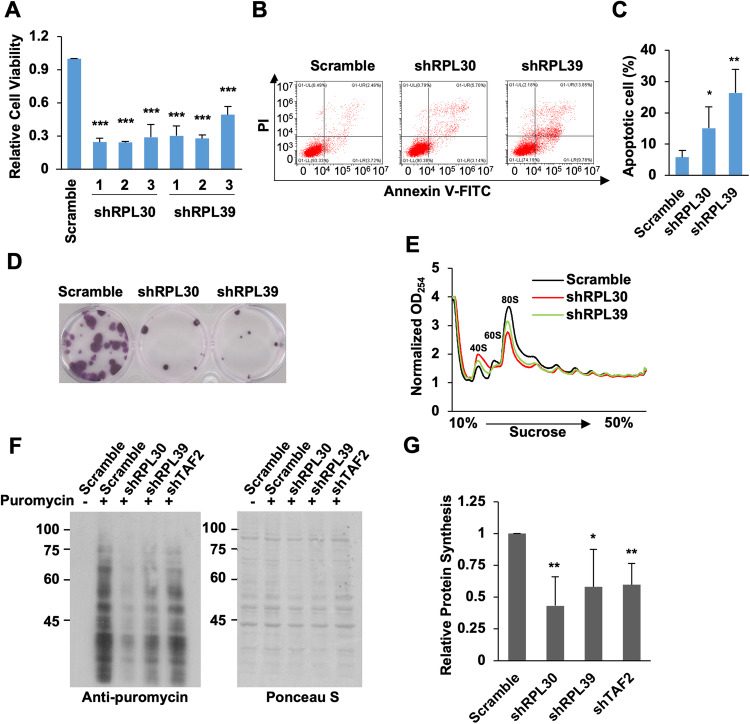
TAF2 and its RP targets are required for ribosome assembly and global protein synthesis. **A** Cell viability assays of HCT116 cells treated with Scramble, RPL30, and RPL39 shRNAs. Results are relative to Scramble and shown as mean ± SD of 3 independent experiments. Student’s *t* test, ***P < 0.001. **B** Representative apoptosis assay of HCT116 cells treated with Scramble, RPL30, and RPL39 shRNAs. **C** Summary of apoptotic cells for HCT116 cells treated Scramble, RPL30, RPL39 shRNAs. Mean ± SD of 3 independent experiments. Student’s *t* test, **P < 0.01; *P < 0.05. **D** Colony-forming assay of HCT116 cells treated Scramble, RPL30, RPL39 shRNAs. **E** Polysome profiles in the cell lysates from HCT116 cells treated with Scramble, shRPL30, and shRPL39. Note: the decrease in area under the 80 S monosome peak and an increase in the 40 S subunit peak is observed in RPL30 or RPL39 depleted cells. **F** Immunoblots of puromycin-labelled proteins (right) in HCT116 cells treated with Scramble, shTAF2, shRPL30, and shRPL39 in the presence or absence of puromycin treatment. Right panel shows the Ponceau S stained membrane for loading control. **G** Plot showing the global protein synthesis in HCT116 cells treated with indicated shRNAs, relative to Scramble. Global protein synthesis was determined by quantitating the overall anti-puromycin reacting signals in **F**. Mean ± SD from 3 independent experiments. Student’s *t* test, **P < 0.01; *P < 0.05.

To investigate the roles of TAF2-dependent direct target genes in cell growth, we identified shRNAs that can efficiently knockdown these genes and carried out cell viability assays (Supplemental Fig. S9). Notably, a decrease of cell viability was observed for cells treated with shRPL30 or shRPL39, but not with other shRNAs (Fig. 6A and Supplemental Fig. S9). Similar to what was observed in TAF2-depleted cells, increased cell apoptosis and decreased colony-forming ability were also detected in HCT116 cells depleted of RPL30 or RPL39 (Fig. 6B–D). Since RPL30 and RPL39 are structural constituents of the 60 S ribosome large subunit, we sought to examine ribosome biosynthesis and protein translation in the cells depleted of TAF2, RPL30, and RPL39. Polysome profiling assays, used to assess the integrity of ribosome and mRNA-engaged polysomes, revealed that the depletion of the ribosomal proteins RPL30 and RPL39 reduced the levels of 60 S, 80 S, and polysomes when compared to the control. In contrast, the levels of the 40 S ribosome small subunit were increased in cells treated with shRPL30 or shRPL39 (Fig. 6E). Interestingly, a similar, but weaker, effect was also observed in shTAF2-treated cells (Supplemental Fig. S10). These observations reveal that the ribosome assembly is impaired in cells depleted of two TAF2-regulated RP genes. To further assess the ribosome function, we employed the “Surface-Sensing of Translation (SUnSET)” assay that offers a convenient alternative to the traditional radioactive labeling method for assessing protein translation efficiency. In SUnSET assay [28], a chase of puromycin treatment was used to label and terminate the elongating polypeptides on ribosomes. The levels of puromycin-labelled polypeptides, indicative of the rate of mRNA translation in the cells, were further analyzed by immunoblotting with an anti-puromycin antibody. Our results showed that the levels of newly-synthesized polypeptides were clearly decreased in TAF2-, RPL30-, or RPL39-knockdown cells (Fig. 6F, G), indicating that TAF2, as well as RPL30 and RPL39, are required for efficient protein synthesis. Overall, our results indicate that TAF2-regulated expression of RPL30 and RPL39 is required for ribosome assembly and global protein synthesis processes that are fundamental for cell proliferation.

## Discussion

The expression of protein-coding genes is central to various biological processes and is tightly regulated by a group of gene-specific regulators and a group of general co-regulators and multicomponent machineries [1, 3]. The multisubunit TFIID complex functions as a general initiation factor by recognizing core promoter elements and facilitating the recruitment and assembly of other GTFs and Pol II into a preinitiation complex (PIC) during the activation of most protein-coding genes [1]. Despite its general initiation functions, several TAF subunits within the TFIID complex have been reported to act in a promoter-specific way [1]. Of special interest here, we combined genome-wide profiling and biochemical analyses to investigate the gene regulatory function of the TAF2 subunit of the TFIID complex. Our Co-IP assays showed that TAF2 interacts with TBP and other TAFs, but appears to be present in only a small fraction of the TFIID complexes in our preparations. Consistent with this notion, our genome-wide profiling assays further indicated that TAF2 binds to, and regulates, TFIID binding to a small subset of Pol II-transcribed genes, particularly genes that encode ribosomal proteins (RPs). Functionally, TAF2 and TAF2-regulated RP genes play important roles in ribosome assembly, which is essential for global protein synthesis and cell proliferation. Therefore, our study suggests that TAF2 is an essential factor for cell growth by directing promoter-specific TFIID binding. However, the TAF2 integration within the TFIID complex and the molecular mechanism for TAF2-mediated TFIID binding to RP genes require further rigorous biochemical and cell-based analysis.

### TAF2 regulates a small fraction of TFIID- and Pol II-bound genes

It is generally believed that the evolutionary conserved TFIID is a stable complex composed of TBP and 13-14 TAFs [10] and is critical for Pol II-transcribed genes, including both protein-coding and non-coding RNA genes. However, biochemical and cell-based analyses also revealed specific TAF dependencies for several activator-mediated transcription events [5, 6, 8]. These findings suggest that certain TAFs within the TFIID complex, through direct TAF-activator interactions, can regulate gene transcription in a promoter-selective way. Previous structural and biochemical analyses of human and yeast TFIID complexes strongly suggest that TAF2 primarily mediates, together with TAF1, the TFIID binding to the Initiator element [12, 13, 15, 29] and interacts with TAF8-TAF10 [30] and TAF14 [31, 32] to stabilize TFIID. These observations suggested that TAF2 may play an essential function in TFIID integrity and chromatin binding. Although previous biochemical analyses revealed that TAF2 is stably associated with the human TFIID complex [14], our Co-IP assays showed that TAF2 is present in only a small fraction of the TFIID complexes, as it was barely detected in the anti-TAF4 immunoprecipitates. In support of this notion, early gel filtration analyses also indicated that a TAF2 (CIF150/TAF_II_150)-free TFIID complex was observed in HeLa nuclear extract [20].

Although it is possible that the labile association of TAF2 in TFIID is easily disrupted during stringent biochemical preparations, our ChIP-seq analysis showed that TAF2 occupies only a small subset (~10%) of TAF3/TFIID- and Pol II-bound gene promoters. The small number of observed TAF2 peaks appears not to be due to the low efficiency of the ChIP-seq assay, as a dramatic difference in TAF2 ChIP-seq signals on several adjacent genes was observed (Fig. 3E). In addition, unlike various biochemical isolations of the TFIID complex, formaldehyde crosslinking in ChIP-seq assays can preserve potentially labile interactions between DNA-binding factors and chromatin. Thus, our results suggest a promoter-selective binding of TAF2 that may contribute to promoter-specific TFIID occupancy. In support of this idea, TAF2 ablation, by AID-mediated degradation within 6-h, significantly reduced the TBP/TFIID occupancy in TAF2-bound (including RP) genes without affecting (or even slightly enhancing) occupancy of genes not normally bound by TAF2 (Fig. 4H). Consistent with the above observation, TAF2 depletion by shRNA-mediated silencing resulted in the downregulation of 1857 genes. Notably, the majority of the dysregulated genes are not bound by TAF2, indicating an indirect effect of prolonged TAF2 loss or a possibility that the remaining TAF2 proteins in the shRNA-treated cells might be sufficient for many TAF2-bound promoters. In support of this notion, microarray analyses revealed that only 3% of the yeast genome is dependent on TAF2 for transcription [9], particularly genes that encode ribosomal proteins. Although a recent study revealed that ablation of yeast TAFs, such as Taf1, Taf2, Taf7, Taf11, and Taf13, decreased nearly all Pol II-dependent nascent transcripts, the Taf2 dependency for either global Pol II binding or transcription was the least affected [22]. Overall, these observations suggest a function for TAF2 in regulating promoter-specific TFIID binding, as best exemplified by binding to RP gene promoters.

### TAF2 selectively regulates genes involved in ribosome assembly that control global protein translation and cell growth

Our cell-based assays have clearly demonstrated a TAF2 dependency for the growth and survival of multiple cancer lines. Consistent with this notion, inactivation of yeast Taf2 also prohibited cell growth [9]; and a recent shRNA screening assay also indicated an essential role of TAF2 in the growth of normal and leukemic murine myeloid cells [8]. In relation to the limited number of direct target genes regulated by TAF2, we focused on two genes encoding ribosomal proteins, RPL30 and RPL39, that are involved in ribosome assembly and global protein synthesis. It is noteworthy that accumulated studies have established a strong link between TFIID activity and RP gene activation [33–36]. In human cells, the 80 S ribosome contains four ribosomal RNAs (rRNAs) and 80 ribosomal proteins (r-protein) organized into two subunits [37]. The 60 S subunit consists of three rRNAs and 47 r-proteins (RPLs in the large subunit), while the 40 S subunit consists of one rRNA and 33 r-proteins (RPSs in the small subunit). Perturbation of ribosome assembly is most likely to impair global protein translation capacity and lead to growth defects or apoptosis of cells [38].

It is notable that our cell-based analyses demonstrated disturbed ribosome assembly and reduced efficiency of global protein translation in cells depleted of TAF2-regulated RPL30 and RPL39, as well as TAF2. In support of this notion, a recent study revealed that the depletion of RPL5 and RPL11 also decreased cell proliferation caused by impaired global protein translation [39]. In addition, the downregulation of RPs has also been reported to activate p53-mediated apoptosis [40, 41] or elevate oxidative stress [23, 42], leading to p53-independent apoptosis, of mammalian cells [43]. Notably, our RNA-seq results revealed that TAF2 depletion significantly increased the expression of KEAP1-Nrf2-dependent genes, including hemp oxygenase-1 (*HXMO1*) and NAPDH quinone oxidoreductase 1 (*NQO1*), which are responsible for various cellular stresses that include oxidation and electrophilic stress [44]. These results suggest that TAF2 plays an important role in ribosome assembly and function by regulating the expression of two ribosomal protein genes, RPL30 and RPL39.

In this work, we established a previously unappreciated pathway in which TAF2 manifests a regulatory role through promoter-specific TFIID binding to RP genes, whose products subsequently regulate the ribosome assembly and global protein translation that is required for cell growth/proliferation.

## Materials and methods

### Antibodies

The anti-TAF2 antibody used in ChIP-seq, immunoprecipitation, and immunoblotting assays was generated with a GST-tagged human TAF2 fragment (aa 920-1108) in rabbits and antigen-specifically purified. Other antibodies are listed in Supplemental Table S1.

### Cell Culture

HCT116 cells were cultured in McCoys’5 A medium (Invitrogen). H1299, A2780, and HEK-293T cell lines were cultured in DMEM medium (Invitrogen). H1975 cells were cultured in RPMI medium (Invitrogen). All cell lines were maintained in media supplemented with 10% fetal bovine serum (FBS) and 1% penicillin-streptomycin (PS) and grown at 37 °C and 5% CO_2_.

### RT-qPCR and RNA-seq assays

The quantitative RT-PCR and RNA-seq assays were carried out as previously described [45]. In brief, total cellular RNAs were isolated from cells treated with the indicated shRNAs for 96 (RT-qPCR) or 144 (RNA-seq) hours using the Quick-RNA Mini-prep kit (Zymo Research) according to the manufacturer’s instructions. For RT-qPCR assays, purified RNAs were reverse-transcribed using the RevertAid First Strand cDNA Synthesis Kit (Thermo Fisher). RNA levels were determined by quantitative real-time PCR with specific primers and KAPA SYBR FAST qPCR Master Mix (KAPA Biosystems) in a StepOnePlus PCR system (Thermo Fisher). Gene expression levels were normalized against *GAPDH* levels. Gene-specific primers used in this study are listed in Supplemental Table S2. For RNA-seq assays, multiplexed RNA-seq libraries were prepared using the Illumina Stranded Total RNA Prep with Ribo-Zero Plus and sequencing by a NextSeq 500 instrument (Illumina) according to the manufacturer’s instructions (Genomic core at the Institute of Molecular Biology, Academia Sinica). Raw RNA-seq data were aligned to human genome assembly HG19 and processed on the Galaxy platform using RNA STAR (2.7.8a+galaxy1), featureCounts (2.0.1+galaxy2), and DEseq2(2.11.40.7+galaxy2) software with default parameters. Total reads, aligned reads, and mapping efficiencies of the RNA-seq samples can be found in Supplemental Table S3.

### Nuclear extract preparation

To prepare nuclear extracts, 1 × 10^8^ cells were detached, washed with ice-cold PBS, and pelleted by centrifugation at 200 × *g* for 10 min at 4 °C. Cell pellets (~0.3 mL) were resuspended in 1.5 mL (5 packed cell volume) hypotonic lysis buffer (10 mM HEPES-KOH pH 7.5, 1.5 mM MgCl_2_, 10 mM KCl, 1 mM PMSF, 5 mM 2-mercaptoethanol, 1× Complete protease inhibitors [Roche]) and incubated on ice for 15 min followed by the addition of 0.3% Triton X 100 (TX100) for 1 min to disrupt the plasma membrane. Cell nuclei were immediately pelleted by centrifugation at 2000 × *g* for 10 min at 4 °C, resuspended in 0.4 mL (2 packed nuclei volume) nuclear extract buffer (20 mM HEPES-KOH pH 7.5, 1.5 mM MgCl_2_, 420 mM KCl, 0.2 mM EDTA, 25% glycerol, 1 mM PMSF, 5 mM 2-mercaptoethanol, 1× Complete protease inhibitors [Roche]) and incubated on ice for 30 min. Nuclear extracts were cleared by centrifugation at 20,000 × *g* for 15 min at 4 °C, and the supernatant was mixed with hypotonic lysis buffer in a ratio of 1:2 to reduce the salt concentration to ~150 mM and snap frozen before use. For whole cell lysates, 1 × 10^7^ cells were detached and resuspended in 1 mL BC300 buffer (20 mM Tris-HCl pH 7.9, 10% glycerol, 0.2 mM EDTA pH 8.0, 300 mM NaCl, 0.2% TX100, 1× Complete protease inhibitor [Roche]) and incubated on ice for 20 min. Cell lysates were cleared by centrifugation at 20,000 × *g* at 4 °C for 15 min and stored at −80 °C before use.

### Immunoprecipitation (IP) and Co-IP

IP and Co-IP assays were carried out as previously described [46]. Briefly, whole cell lysates of 1 × 10^7^ cells or 1 mg nuclear extracts were incubated with ~2 μg indicated antibody and 10 μL magnetic Protein G beads (Invitrogen) at 4 °C for 1~2 h. Beads were washed 3 times with BC150 buffer (20 mM Tris-HCl pH 7.9, 10% glycerol, 0.2 mM EDTA pH 8.0, 150 mM NaCl, 0.2% TX100) and denatured in 1× sample buffer at 95 °C for 5 min. The eluted protein samples were separated by gel electrophoresis and analyzed by immunoblotting.

### shRNA knockdown

All shRNA clones were obtained from the National RNAi Core Facility at Academia Sinica in Taiwan and are listed in Supplemental Table S4. Lentiviruses were prepared according to a standard protocol from Addgene and as previously described [45].

### Cell proliferation, cell counting kit-8 (CCK-8), and colony-forming assays

The shRNA-treated cells were selected with puromycin for 48 h and seeded in 6-well plates. For proliferation assays, 50,000 cells/well were seeded and live cells were counted with trypan blue on the indicated days. For colony-forming assays, 2000 cells/well were seeded, and the medium was changed every 3 days. After 10~14 days, cells were fixed with 4% formaldehyde (in PBS) and stained with a crystal violet solution (PBS containing 0.05% crystal violet, 1% formaldehyde, and 1% methanol) for 1 h. After being extensively washed with ddH_2_O, the images of colonies were scanned with an optical scanner. For CCK-8 assays, 3000 cells/well were seeded on 96-well plates, and CCK-8 solution (DUDB4000X, Dojindo Molecular Technologies) was added to cells after 3 days incubation for 2 h. OD450 was determined in an i-control^TM^ Microplate Reader (TECAN).

### Cell cycle and apoptosis assays

The shRNA-treated cells were selected with puromycin treatment for 48 h and incubated on culture dishes for the indicated time. For cell cycle assays, cells were fixed with 70% ethanol and stained with a PI solution (20 μg/mL PI, 0.1% Triton-X 100, and 0.2 mg/mL RNase A). For apoptosis assays, cells were stained directly with Annexin A5 (ab14082) and PI (1 μg/mL). Results were analyzed in a CytoFLEX flow cytometer (Beckman), and cell cycle profiles were analyzed using FlowJo software.

### ChIP-qPCR, ChIP-seq assays, and data analysis

ChIP-qPCR and ChIP-seq assays were carried out as previously described [45]. Briefly, cells were cross-linked with 1% formaldehyde and quenched with 125 mM glycine. Chromatin lysates were prepared in FA lysis buffer (50 mM HEPES-KOH pH 7.5, 140 mM NaCl, 1 mM EDTA pH 8.0, 1% Triton X100, 0.1% sodium deoxycholate, 0.1% SDS, 0.25% sarcosyl, 1× Complete protease inhibitors) and sonicated with a Bioruptor pico (4 times, high output, 30 sec ON/30 sec OFF). After immunoprecipitation with indicated antibodies, samples were treated with RNase A and Protease K. The ChIPed genomic DNAs were purified using the PCR purification kit (Qiagen) and subjected to quantitative PCR with indicated gene primers or to library construction and high-throughput DNA sequencing on an Illumina HiSeq 2000 according to the manufacturer’s protocol. Raw sequencing data were examined by FastQC (v0.11.5) and aligned to human genome assembly HG19 using bowtie2 (2.5.0+galaxy0) with the default parameters on the Galaxy platform. Duplicates were removed using *rmdup* of SAMtools (v1.9). The number of total reads, uniquely aligned reads, and mapping efficiencies are listed in Supplemental Table S5. Peak finding was performed using MACS (1.4.2). Peak overlap analyses were performed using HOMER suite (v4.9.1) as previously described [45], and lists of identified ChIP-seq peaks can be found in the Supplementary materials.

### Generating TAF2-AID HCT116 cell line

To insert the plant-specific minimal auxin-inducible degron (mAID) tag at the c-terminus of the endogenous TAF2 locus in HCT116 cells, the pGEMT-TAF2-donor-288 was constructed by ligations of a PCR-amplified cDNA sequence of mAID-Bsr of pMK288 (Addgene #72826) and PCR-amplified exon 26 of TAF2 (Supplemental Table S2). HCT116 cells were co-transfected with pGEMT-TAF2-donor-288 and eSpCas9_1.1_TAF2_gRNA (target sequence: GAATAGACCTGCCACTGGCA) constructs using PolyJet reagent (SignaGen). Positive knock-in clones were screened by genotyping and transduced with Lentiviruses expressing the reverse tetracycline-transactivator 3 (rtTA3), pTRIPZ-rtTA3 [47], and OSTIR1-myc-mcherry. The PL-SIN-5TO-NLS-OSTIR1-myc-mCherry was constructed by inserting the OSTIR1-3Myc fragment of pBabe-Puro-osTIR1-9Myc (Addgene #80074) into *Bam HI* sites of linearized PL-SIN-5TO-NLS-IRES-mCherry [47]. After selection with puromycin treatment for 2 weeks and isolation with the mCherry signal by FACSorting, AID-TAF2 Clone #28 was used in the following experiments. For the TBP ChIP-seq experiment, AID-TAF2 Clone #28 cells were pretreated with 1 μM doxycycline for 36 h, followed by incubation with 1 μM doxycycline and 0.5 mM IAA for indicated times, and cells were collected for ChIP assay with anti-TBP antibody.

### Genotyping for TAF2-mAID clones

Cells were lysed with DNA Lysis buffer (100 mM Tris-HCl [pH 8.0], 200 mM NaCl, 5 mM EDTA, 1% SDS, 0.6 mg/mL protease K, 0.3 M NaOAc.), incubated at 55 °C for 1 h and subjected to isopropanol precipitation. After washing with 70% ethanol, the DNA pellet was dissolved in TE buffer containing 50 μg/mL RNase A and incubated at 65 °C for 1 h. The isolated genomic DNA (1 μg) was PCR-amplified with KAPA Taq ReadyMix and the indicated primers: WT-F, WT-R, AID-R, and AID-F (Supplemental Table S2). The PCR program was 95 °C 3 min, (95 °C 1 min, 56 °C 1 min, 72 °C 1 min) for 40 cycles, and 72 °C 5 min. The PCR products were analyzed by agarose gel electrophoresis.

### Polysome profiling

The shRNA-treated cells were incubated with 100 μg/mL cycloheximide (CHX) for 8 min at 37 °C. Cells were then detached and resuspended in a hypotonic buffer (5 mM Tris-HCl [pH 7.5], 2.5 mM MgCl_2_, 1.5 mM KCl, 100 μg/mL CHX, 1 mM DTT, 100 U RNase inhibitor, 0.5% Tx100, 0.5% sodium deoxycholate, 1× Complete protease inhibitor cocktail (EDTA-free)]. After centrifugation at 22,000 × *g*, 4 °C, for 7 min, a fixed amount of cell lysate (16 OD) was loaded onto a sucrose gradient (10-50%) and centrifuged at 222,000 × *g*, 4 °C for 3 h in a Beckman SW41 Ti swing-bucket rotor. Polysome profiles were monitored by OD254 in a Density Gradient Fractionation System (Brandel BR-188).

### Surface-sensing translation (SUnSET) assay

The SUnSET assays were carried out as previously described [28]. Briefly, HCT116 cells, 48 h post-infection with the indicated shRNAs, were seeded in 24-well plates at a density of 1.2 × 10^5^/well for 24 h. Cells were treated with 2.5 μg/mL puromycin for 10 min at 37 °C. After washing with ice-cold PBS, cells were directly lysed and denatured in 1× Laemmli sample buffer. Samples were separated by gel electrophoresis and subjected to immunoblotting with anti-puromycin. The optical densities of puromycin-labelled proteins were determined by ImageJ software.

### Statistics and reproducibility

All experiments with representative images (such as immunoblot, Co-IP, and colony-forming assays) were independently repeated at least three times with similar results. Presented plots are shown as mean ± SD of three biological experiments. The differences were assessed by Student’s *t* test, and *p* values are denoted as follows: *P < 0.05; **P < 0.01; ***P < 0.001. For TBP occupancy, the nonparametric Mann–Whitney U test was performed, and ***P < 0.001 were denoted.

### Supplementary information

Supplemental Data

Original Immunoblot Images

Dataset 1

## Data Availability

Sequencing (ChIP-seq and RNA-seq) data generated in this study have been deposited in the NCBI Gene Expression Omnibus (GEO) database under accession number GSE232675.
