# Supplementary material for: TAF2, within the TFIID complex, regulates the expression of a subset of protein-coding genes
Source: Cell Death Discov. 2024 May 21;10:244. doi: 10.1038/s41420-024-02017-z (PMC11109217; doi:10.1038/s41420-024-02017-z)
Supplement: Supplementary file 1 — Supplemental Data [file 41420_2024_2017_MOESM1_ESM.docx]

**Supplementary Data**

**
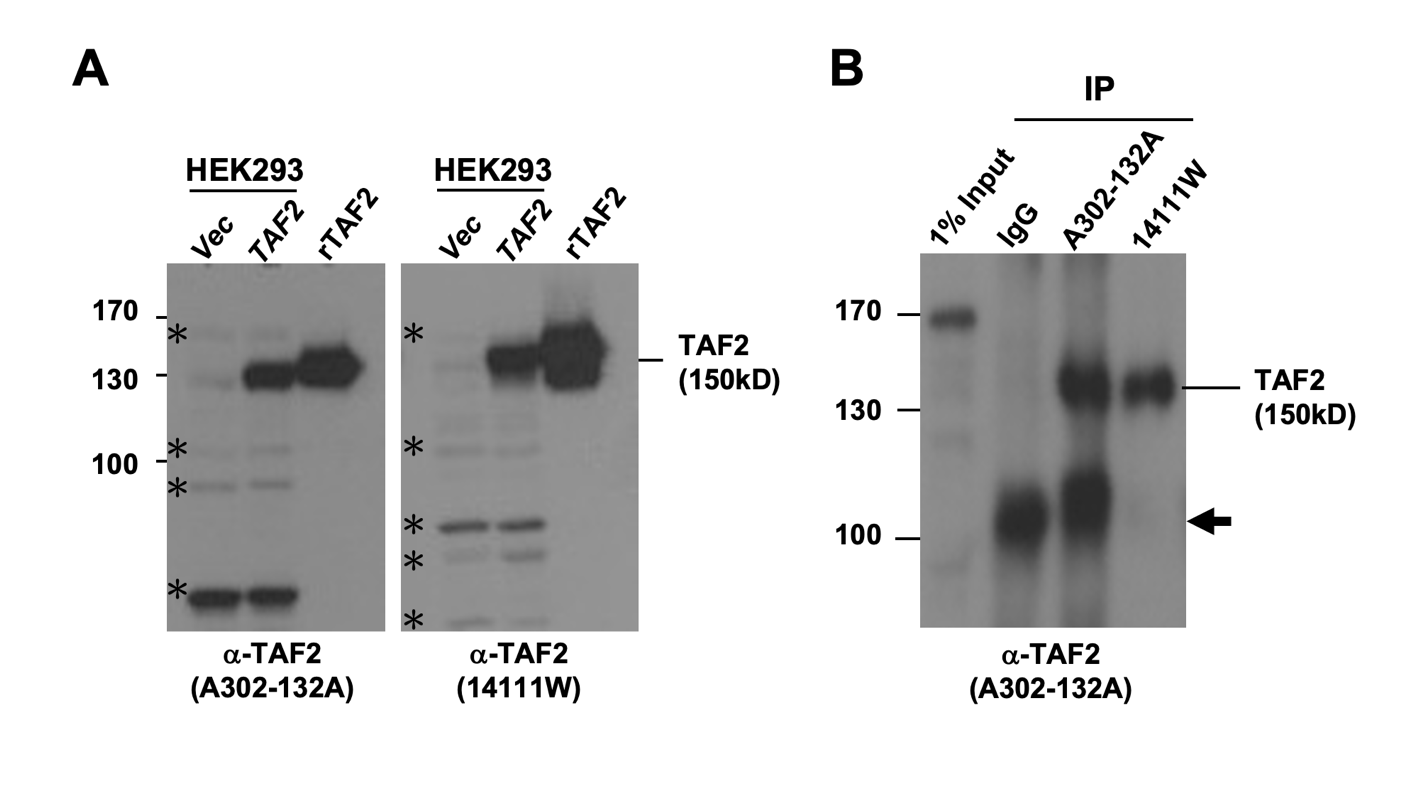
**

**Supplemental Figure S1. Validation of homemade anti-TAF2 antibody (14111W) in immunoblotting and immunoprecipitation assays**

1. Immunoblots of TAF2 proteins using commercial (left, Bethyl A302-132A) or homemade (right, 14111W) anti-TAF2 antibodies in the whole-cell lysates prepared from HEK-293 cells transfected with empty (vec) or TAF2 expression vector (TAF2). Recombinant TAF2 protein (rTAF2, 20 ng) serves as a positive control. Stars denote non-specific signals.
2. Immunoprecipitation assay showing the specificity of commercial (A302-132A) and homemade (14111W) anti-TAF2 antibodies. Whole-cell lysates were prepared from HEK-293 cells and subjected to immunoprecipitation using normal rabbit IgG (IgG), commercial (A302-132A), and homemade (14111W) anti-TAF2 antibodies. Immunoblot was probed with a commercial anti-TAF2 antibody (A302-132A). Normal rabbit IgG serves as a control. The arrow denotes non-specific signals.

**β-tubulin**


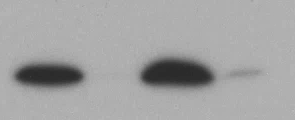

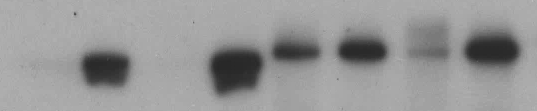


**C**

**N**

**C**

**N**

**C**

**N**

**C**

**N**

**HEK293**

**HCT116**

**HEK293**

**HCT116**

**1% Input**

**TAF4 IP**


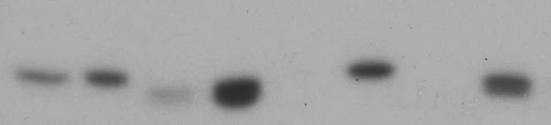


**TAF4**

**TBP**


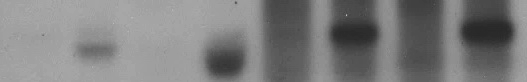


**TAF6**


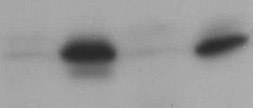


**Lamin B1**

**180**

**140**

**75**

**45**

**60**

**45**

**75**

**60**

**Supplemental Figure S2. Nuclear, but not cytoplasmic, TAF4 associates with the TAF6 and TBP subunits of TFIID complex.**

Co-immunoprecipitation assays showing TAF4 interaction with TAF6 and TBP in the nuclear, but not cytosolic, extracts from the HEK293 and HCT116 lines. Immunoprecipitations (IP) were performed with anti-TAF4 antibody and cytosolic (C) or nuclear (N) extracts prepared by a method for a small-scale preparation of nuclear extracts (details described in Material and Methods). The β-tubulin and Lamin B1 are markers for cytosolic and nuclear extracts, respectively.

**
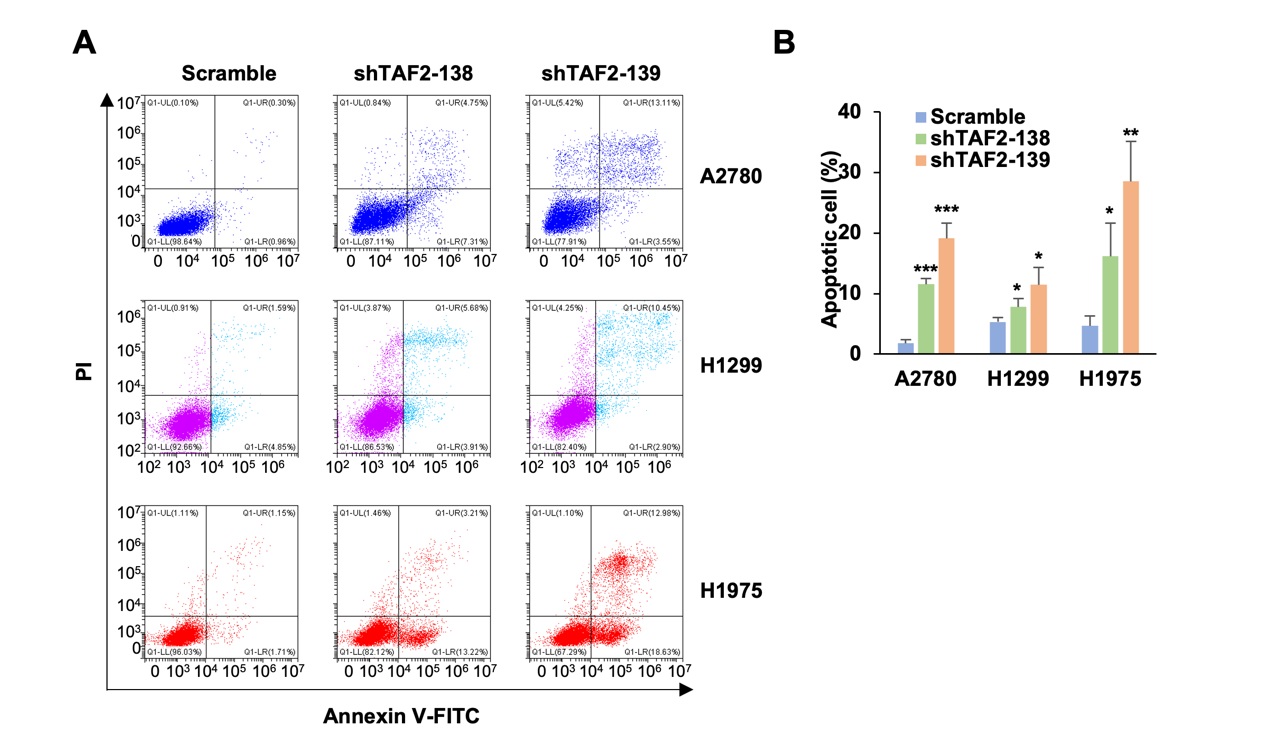
**

**Supplemental Figure S3. Depletion of TAF2 induced cell apoptosis in cancer cell lines.**

1. Flow cytometry analyses of staining for Annexin and DNA content (Propidium Iodide, PI) in ovarian A2780 and lung H1299 and H1975 cancer cell lines treated with Scramble or TAF2 (shTAF2-138 and shTAF2-139) shRNAs.
2. Quantification of apoptotic cells in panel (A). Mean ± SD of 3 biological experiments. Student’s t-test, *, *P* <0.05, **, *P* <0.01, ***, *P* <0.001.


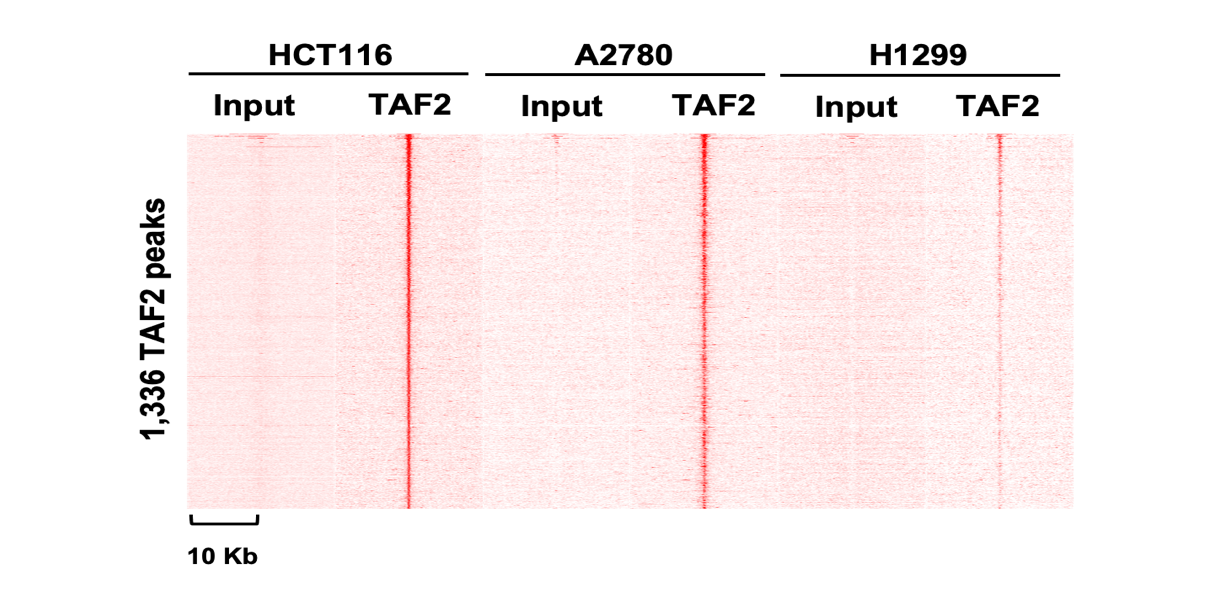


**Supplemental Figure S4. Genomic profiles of TAF2 in cancer cell lines.**

Heatmap showing the input DNA and TAF2 ChIP-seq signals in colorectal HCT116, ovarian A2780, and lung H1299 cancer cell lines. Plots are centered at 1,336 TAF2 ChIP-seq peaks identified in HCT116 cells.

**
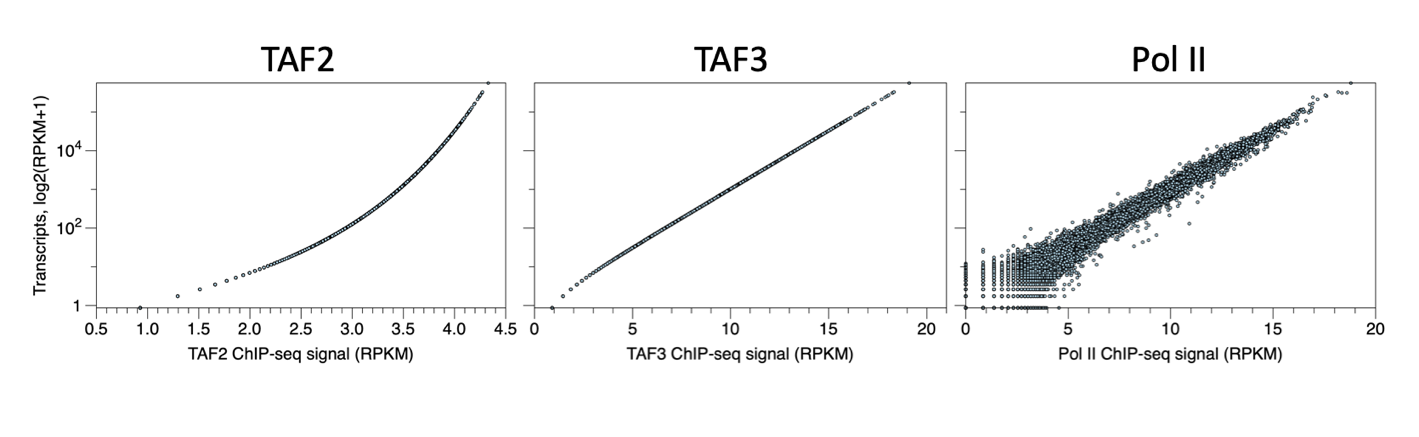

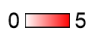

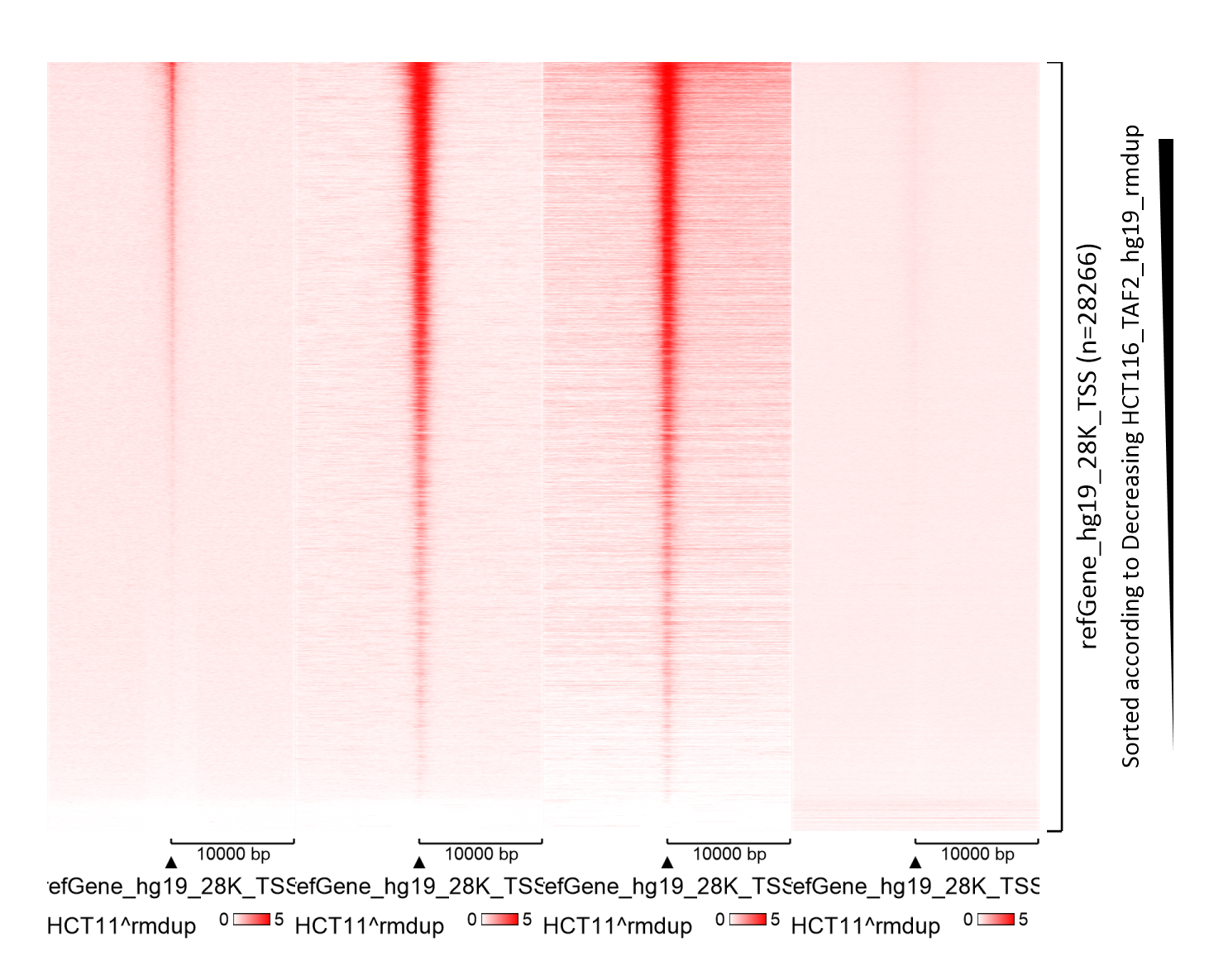
**

**B**

**A**

**input**

**Pol II**

**TSS of hg19 Ref.Genes (28,266)**

**10 Kb**

**10 Kb**

**10 Kb**

**10 Kb**

**TAF2**

**TAF3**

**Supplemental Figure S5. Genomic profiles of TAF2, TAF3, and RNA Pol II in HCT116.**

1. Heatmap showing TAF2, TAF3, and RNA Pol II ChIP-seq signals in colorectal HCT116 cell line. Plots are centered at the transcription start sites (TSS) of 28,266 hg19 reference genes (Ref.Genes). The color key is shown at the bottom.
2. Plots showing the level of transcripts relative to the ChIP-seq signals of TAF2, TAF3, and RNA Pol II in HCT116 cells.

**
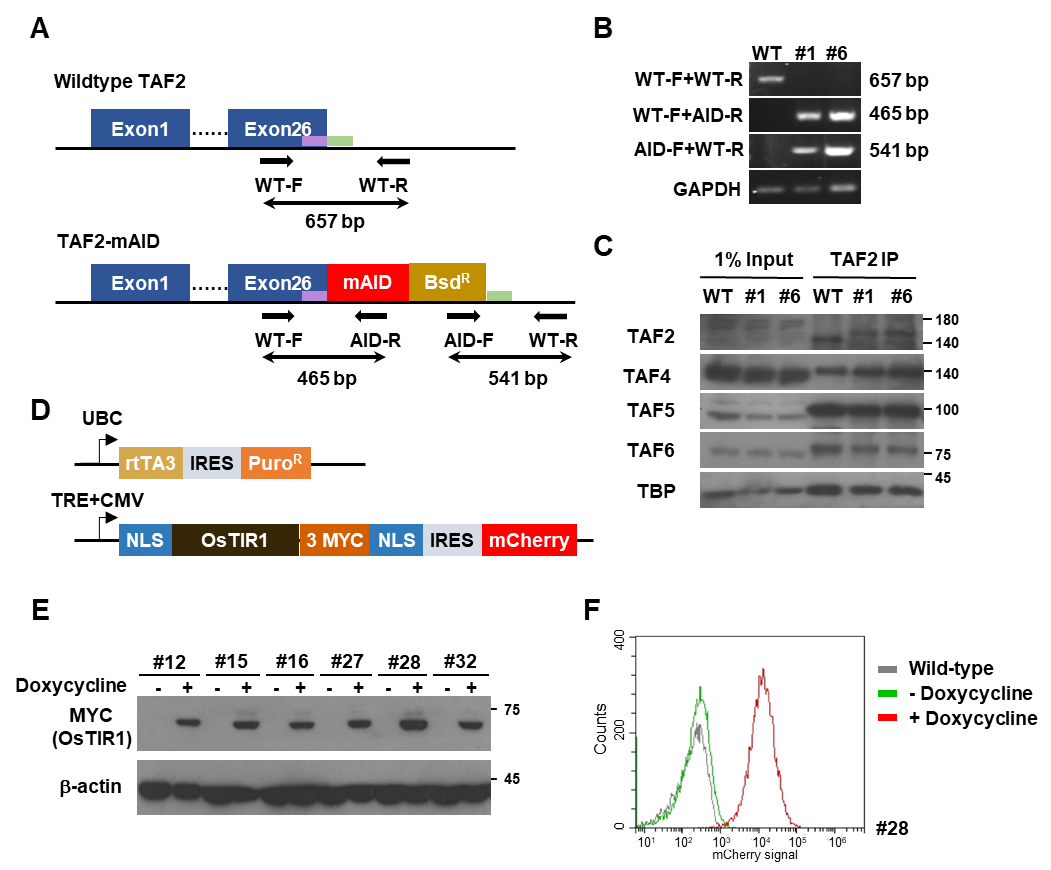
**

**Supplemental Figure S6. Establishment of Auxin-inducible degradation TAF2 system in HCT116 line.**

1. Schematic representation of *TAF2* gene and the primers used for genotyping. mAID, mini-Auxin-inducible degron; Bsd^R^, Blasticidin resistance gene.
2. Genotyping of TAF2-mAID knock-in HCT116 cell lines (#1, #6). Agarose gel showing the DNA fragments amplified by the indicated primers. GAPDH locus was as a control.
3. Co-immunoprecipitation assays showing TFIID integration by mAID-fused TAF2. Immunoprecipitations were performed with anti-TAF2 antibody and cell lysates of wild-type or TAF2-mAID knock-in (#1 and #6) HCT116 cells. Wild-type and mAID-fused TAF2 are indicated on the right; stars on the left indicate nonspecific bands.
4. Schematic of expression constructs for rtTA3 and OsTIR1. The expression of rtTA3-IRES-puromycin is under the control of the human Ubiquitin C promoter. The expression of OsTIR1-IRES-mCherry is under the control of TRE elements and a mini-CMV promoter.
5. Immunoblots of OsTIR1 expression (by anti-Myc-tag antibody) in isolated TAF2-mAID knock-in HCT116 stably inducible OsTIR1 expression clones (AID-TAF2 HCT116 clones) in the presence or absence of doxycycline. Beta-actin is a loading control.
6. Flow cytometry analyses of the mCherry signal (represented for OsTIR1 expression) of the AID-TAF2 HCT116 clone #28 treated without (green) or with (red) doxycycline. Wild-type HCT116 (grey) is a negative control. Note: the mCherry signal can be detected in ~99% AID-TAF2 HCT116 cells.


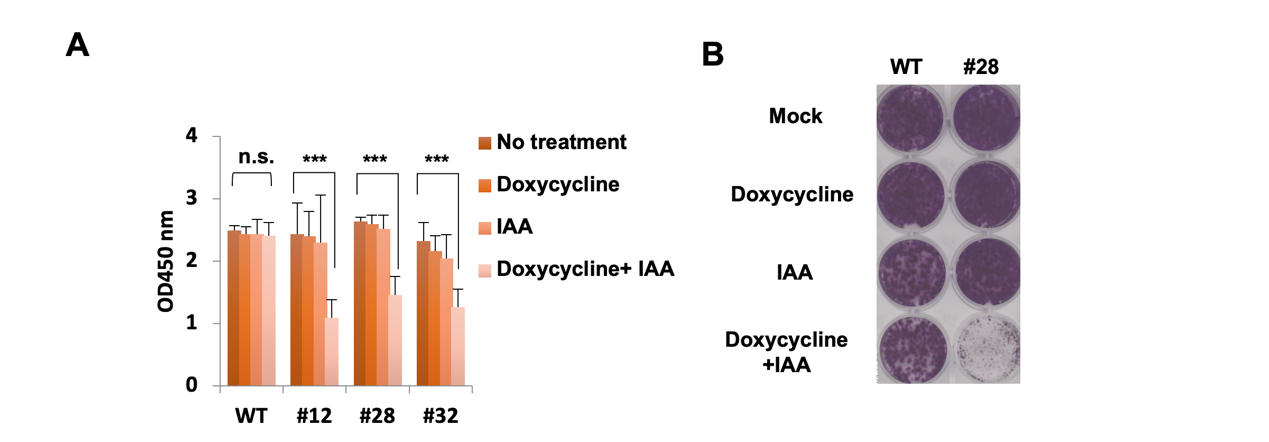


**Supplemental Figure S7. Treatment with doxycycline and auxin repressed the growth of the AID-TAF2 HCT116 clone.**

1. Cell viability assays of wild-type (WT) and AID-TAF2 HCT116 clones (#12, #28, #32) treated as indicated for 72 hours. Shown are the results of cell counting kit-8 (CCK-8) assay. Student’s t-test, ***, *P* <0.001; n.s., not significant.
2. Representative result of the colony-forming assay of wild-type (WT) and AID-TAF2 (#28) HCT116 lines treated with indicated for 10 days. Crystal violate stained images of cells are shown.

**138**

**1495**

**631**

**24h IAA**

**(836)**

**6h IAA**

**(2,214)**

**53**

**35**

**953**

**14**

**TAF2-bound**

**(1055)**

**Genes with increased TBP ChIP-seq signal**

**Supplemental Figure S8. Overlaps of genes with increased TBP ChIP-seq signals in 6 h and 24 h- IAA treatment and TAF2-bound.**

Venn diagram showing the overlap between genes with increased TBP ChIP-seq signal in 6 h and 24 h IAA treatment and TAF2-bound. The threshold of increased TBP ChIP-seq was set as fold change ≥ 1.25, relative to control cells.


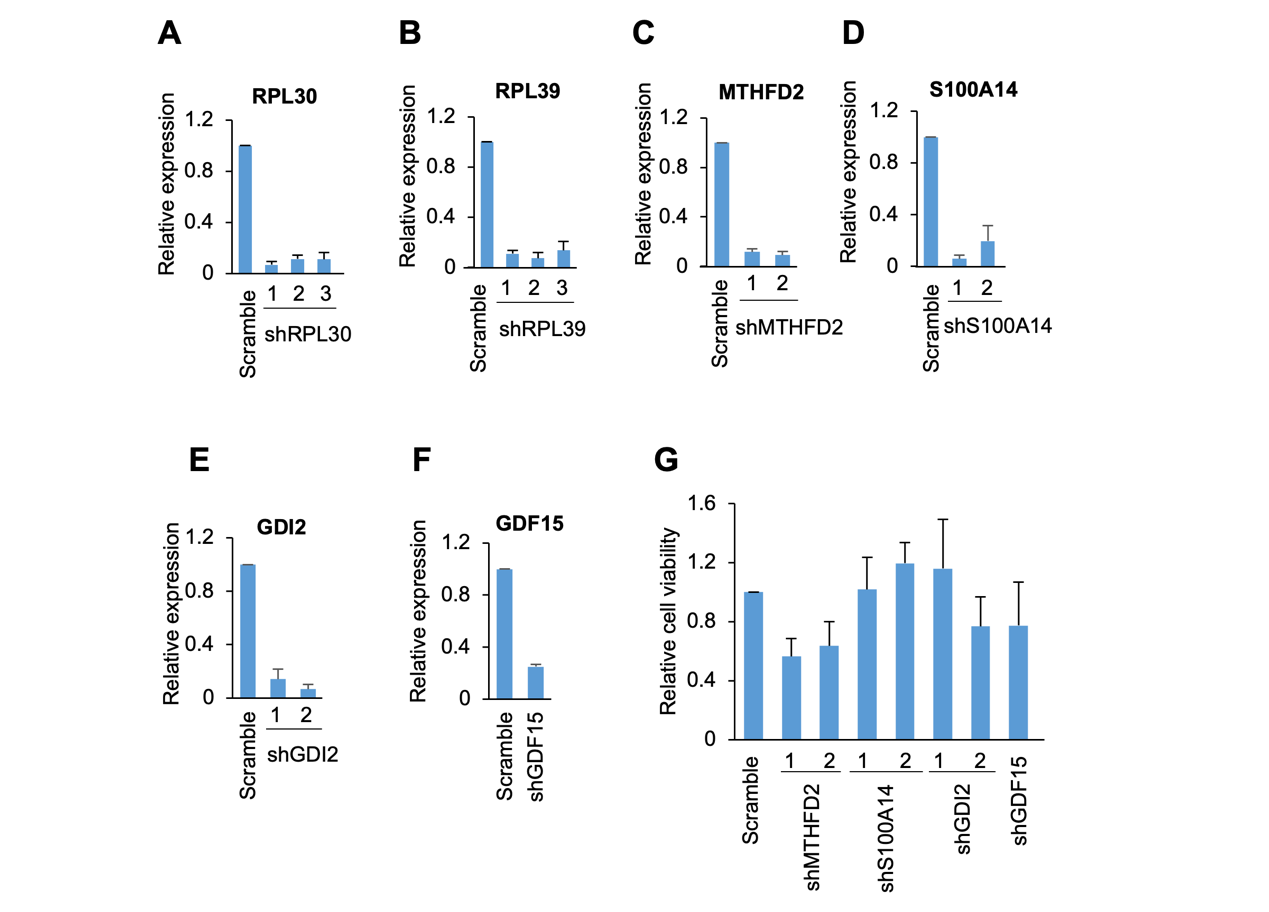


**Supplemental Figure S9. The silence efficiency of shRNAs against indicated TAF2 target genes and their effects on cell viability.**

A-F. RT-qPCR assays showing the mRNA level of the indicated genes in cells treated with shRNAs against RPL30 (A), RPL39 (B), MTHFD2 (C), S100A14 (D), GDI2 (E), or GDF15 (F).

G. Relative cell viability of HCT116 cells treated with Scramble, shMTHFD2, shS100A14, shGDI2, and shGDF15. Cell viability was determined by the cell counting kit-8 (CCK-8) assay.

**
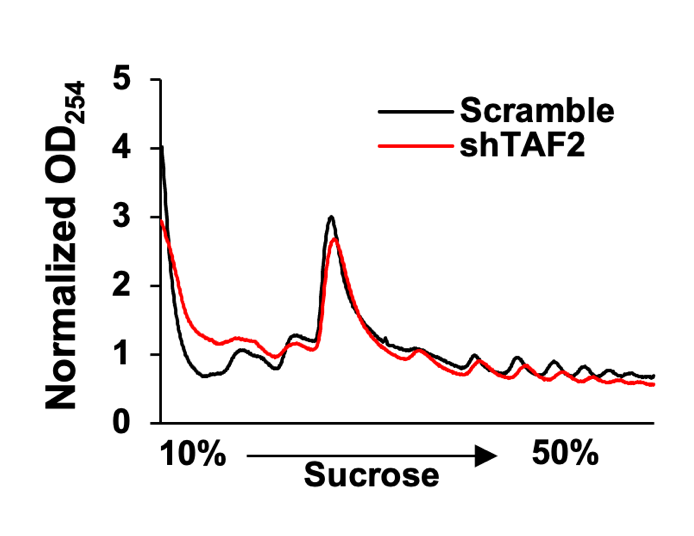
**

**Supplemental Figure S10. Polysome profiles in the lysates of HCT116 cells treated with shTAF2 or shScramble.**

Cell lysates from HCT116 treated with the indicated shRNAs were fractionated in a linear 10~50% sucrose gradient and the absorbance was recorded at 254 nm. The curves of normalized OD254 for cells treated with Scramble (black) and shTAF2 (red) are shown.

**Supplemental Table S1. Antibody list**

| Anti-TAF2 | Raised and purified in this study |
| --- | --- |
| Anti-TAF2 | A302-132A, Bethyl |
| Anti-TAF4 | Roeder lab, The Rockefeller University |
| Anti-TAF5 | Roeder lab, The Rockefeller University |
| Anti-TAF6 | Roeder lab, The Rockefeller University |
| Anti-TAF7 | Roeder lab, The Rockefeller University |
| Anti-TBP (for western) | Roeder lab, The Rockefeller University |
| Anti-TBP (for ChIP-seq) | Ab28175, Abcam |
| Anti-puromycin | MABE343, Merck |
| Anti-Myc | Cell Signaling Tech. #2276 |
| H3K4Me3 | Ab8580, Abcam |
| H3K27Ac | Ab4729, Abcam |
| H3K4Me | Ab8895, Abcam |
| H3K18Ac | Ab40888, Abcam |
| β-actin | A5441, Merck |
| β-tubulin | Ab52901, Abcam |
| Lamin B1 | Ab16048, Abcam |

**Supplemental Table S2. Primers for RT-qPCR, ChIP-qPCR, mAID cloning, and TAF2 genotyping**

**RT-qPCR**

|  | Forward | Reverse |
| --- | --- | --- |
| TAF2 | TCAGAACCCTTTTTCCAGTTCTCA | GGAGCTAGCTGGCCGATATT |
| RPL30 | AACTGCCCAGCTTTGAGGAA | TGCCACTGTAGTGATGGACAC |
| RPL39 | GTGTGTTCTTGACTCCGCTG | TCCTTTTGGAGTTGTACCTGAT |
| S100A14 | GCTGACCCCTTCTGAGCTAC | TGCCCAGGTTGGCAATTTTC |
| MTHFD2 | AAACACATCTGTCTGGTATGGT | TGGTTAGGTCACAACTAGGAGTC |
| GDI2 | CTCGTAGGGTGGTCACGGAG | ACAGGATACATTCCGTCAGGC |
| GDF15 | ATTCGAACACCGACCTCGTC | GAGAGATACGCAGGTGCAGG |
| GAPDH | AAGGTGAAGGTCGGAGTCAAC | GGGGTCATTGATGGCAACAATA |

**ChIP-qPCR**

|  | Forward | Reverse |
| --- | --- | --- |
| TAF6_TSS | ACAGACACACAACCAACCGT | GCCGCTAGAGTTTTCCTGCT |
| EEF1G_TSS | CCCGAATCAGTTCCCCACTC | GGGGTAAGTTTGCTGGCTCT |
| RPS6_TSS | CGCCACCATCACCTACCTTC | ATGCTCACTTCCGCTATCCC |
| RPS7_TSS | ACTCTCTAGCCTCCTACCGC | CGAGAGCACGTCAAAATCCG |
| RPS15_TSS | CTGGATGTTGGGGCGAGGG | GTTACGAGGACCCGGACATGA |
| RPL6_TSS | CCCTGTAGGTGTCGACTGGA | TTGCAAGGTAAGAATCGCGG |
| RPL37A_TSS | CGCGTCTCTTCCTTTCTGGG | AGAGAACACGGGGTAAGGCA |
| RPL30_TSS | AATGCAAAGCTCTTCACGGC | CAGCCATTCTAGCTAGCGGT |
| RPL39_TSS | CAAGAACACACCACGATGGC | AAGCGCCTTCTAAGCTCGTT |
| T&S (intergenic control) | TCTGGAACCCTTCTGTCCCA | CCAGCCATCCAACACAAAGC |

**mAID cloning**

| Plasmid |  | Forward | Reverse |
| --- | --- | --- | --- |
| pGEMT-easy-TAF2-donor-288 | Left arm | CAGTCTTTACTAAGGAATCTACAGCC | AAAGGATCCGTCTGAAAGGGAAGGAGAA |
|  | Right arm | AAAGGATCCGTCTATTCGTTCTCCTTCCC | ATGCTTAGAACTTAAATGAATTCAGAATTTCTGTAGGAGAGG |
| eSpCas9 | gRNA for TAF2 | CACCGAATAGACCTGCCACTGGCA | AAACTGCCAGTGGCAGGTCTATTC |

**Genotyping**

| WT-F | GCACCTTTGGAGATGAGTATGCATC |
| --- | --- |
| WT-R | CAGATTTGGCAGTTGCTTCTGTG |
| AID-F | CTAATTCCATCAGAAGCTGGTCGAG |
| AID-R | CATCCTCAAATCGATTTTCCTCAAGTAC |

**Supplementary Table S3.** **Summary of the number of reads and alignment rate in RNA-seq experiments.**

| **RNA-seq experiment** | **Total reads** | **Uniquely aligned reads (%) ^a^** | **Aligned > 1 times (%)^a^** | **Overall alignment rate (%)** |
| --- | --- | --- | --- | --- |
| HCT116_Scr_#1 | 54,387,141 | 47,881,304 (88.04) | 5,973,004 (10.98) | 99.02 |
| HCT116_Scr_#2 | 56,272,758 | 49,793,819 (88.49) | 5,988,792 (10.64) | 99.13 |
| HCT116_shTAF2_#1 | 47,663,801 | 41,715,788 (87.52) | 5,366,065 (11.26) | 98.78 |
| HCT116_shTAF2_#2 | 44,622,294 | 38,770,267 (86.89) | 5,158,309 (11.56) | 98.45 |

^a^ percent total reads

**Supplemental Table S4. Clone ID for shRNAs**

| Gene symbol | NM ID | Clone ID |
| --- | --- | --- |
| TAF2 | NM_003184 | 138, [TRCN0000053138](https://rnai.genmed.sinica.edu.tw/cloneInfo/cloneId/TRCN0000053138) |
|  |  | 139, [TRCN0000053139](https://rnai.genmed.sinica.edu.tw/cloneInfo/cloneId/TRCN0000053139) |
| RPL30 | [NM_000989](http://www.ncbi.nlm.nih.gov/nuccore/NM_000989) | 567, [TRCN0000117567](https://rnai.genmed.sinica.edu.tw/cloneInfo/cloneId/TRCN0000117567) |
|  |  | 568, [TRCN0000117568](https://rnai.genmed.sinica.edu.tw/cloneInfo/cloneId/TRCN0000117568) |
|  |  | 570, [TRCN0000117570](https://rnai.genmed.sinica.edu.tw/cloneInfo/cloneId/TRCN0000117570) |
| RPL39 | NM_001000 | 632, [TRCN0000117632](https://rnai.genmed.sinica.edu.tw/cloneInfo/cloneId/TRCN0000117632) |
|  |  | 635, [TRCN0000117635](https://rnai.genmed.sinica.edu.tw/cloneInfo/cloneId/TRCN0000117635) |
|  |  | 636, [TRCN0000117636](https://rnai.genmed.sinica.edu.tw/cloneInfo/cloneId/TRCN0000117636) |

| **ChIP-seq experiment** | **Total reads** | **Uniquely aligned reads** | **Number of peaks detected**  **P<=10^-9^** | **Mapping Efficiency (%)** |
| --- | --- | --- | --- | --- |
| input A2780 | 29,120,678 | 20,906,477 |  | 71.79 |
| TAF2 A2780 | 23,395,048 | 17,686,341 | 2,065 | 75.60 |
| Input H1299 | 28,259,065 | 14,504,413 |  | 51.33 |
| TAF2 H1299 | 36,866,403 | 25,249,258 | 246 | 68.49 |
| H3K4Me1 HCT116 | 37,860,384 | 30,036,646 |  | 79.34 |
| H3K4Me3 HCT116 | 36,560,547 | 28,107,886 |  | 76.88 |
| H3K18Ac HCT116 | 44,702,959 | 33,240,746 |  | 74.36 |
| H3K27Ac HCT116 | 37,924,714 | 30,505,835 |  | 80.44 |
| Input HCT116 | 39,949,582 | 27,185,794 |  | 60.05 |
| TAF2 HCT116 | 18,963,643 | 14,875,132 | 1,336 | 78.44 |
| TBP HCT116 IAA_0h | 37,176,900 | 26,374,004 |  | 70.94 |
| TBP HCT116 IAA_6h | 41,610,674 | 28,042,015 |  | 67.39 |
| TBP HCT116 IAA_24h | 40,147,491 | 28,000,518 |  | 69.74 |

**Supplementary Table S5. Summary of the number of reads and peaks of ChIP-seq assays.**
