# Supplementary material for: TAF2, within the TFIID complex, regulates the expression of a subset of protein-coding genes
Source: Cell Death Discov. 2024 May 21;10:244. doi: 10.1038/s41420-024-02017-z (PMC11109217; doi:10.1038/s41420-024-02017-z)

Figure 1A (HCT116)

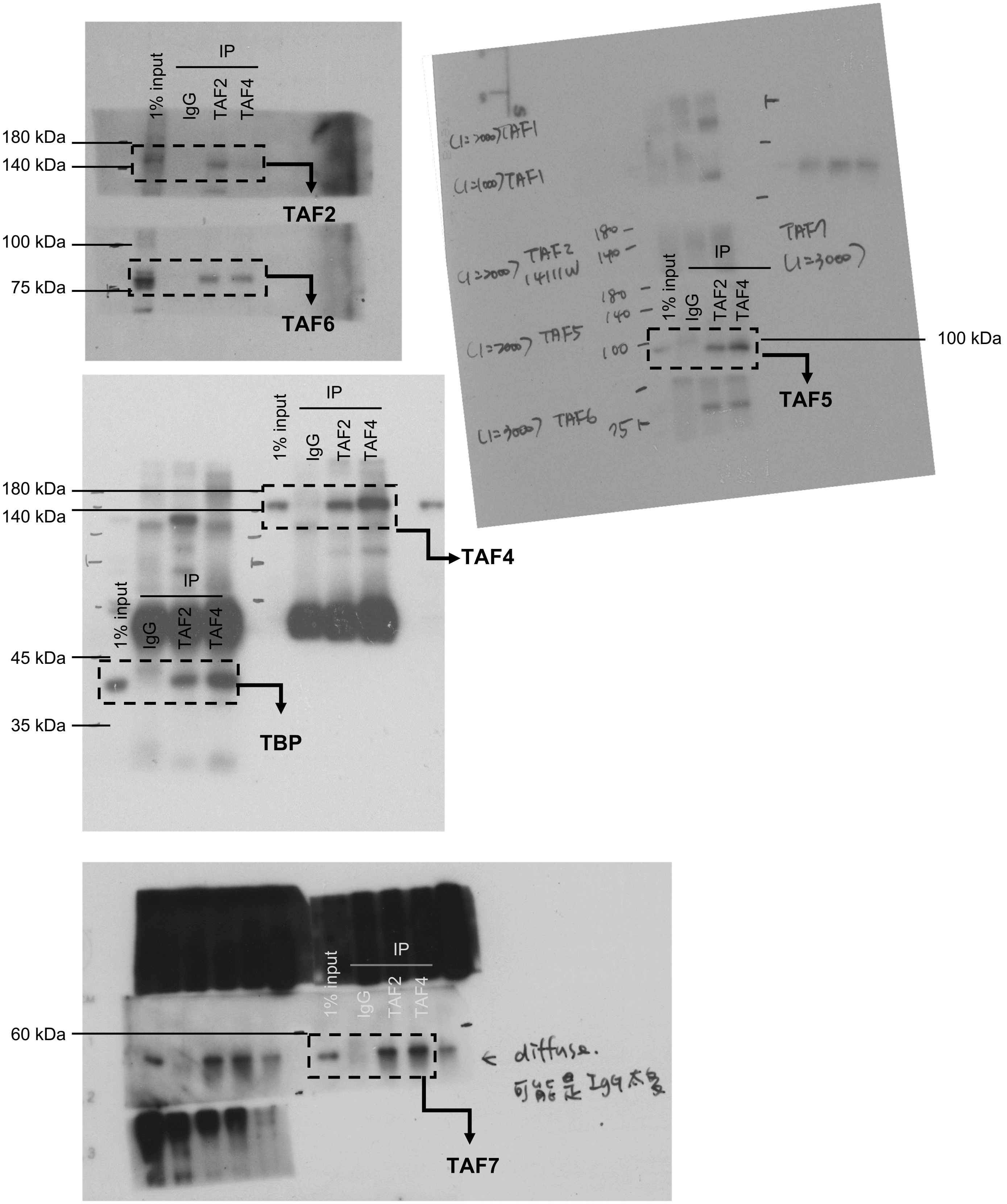

Figure 1A (HEK-293)

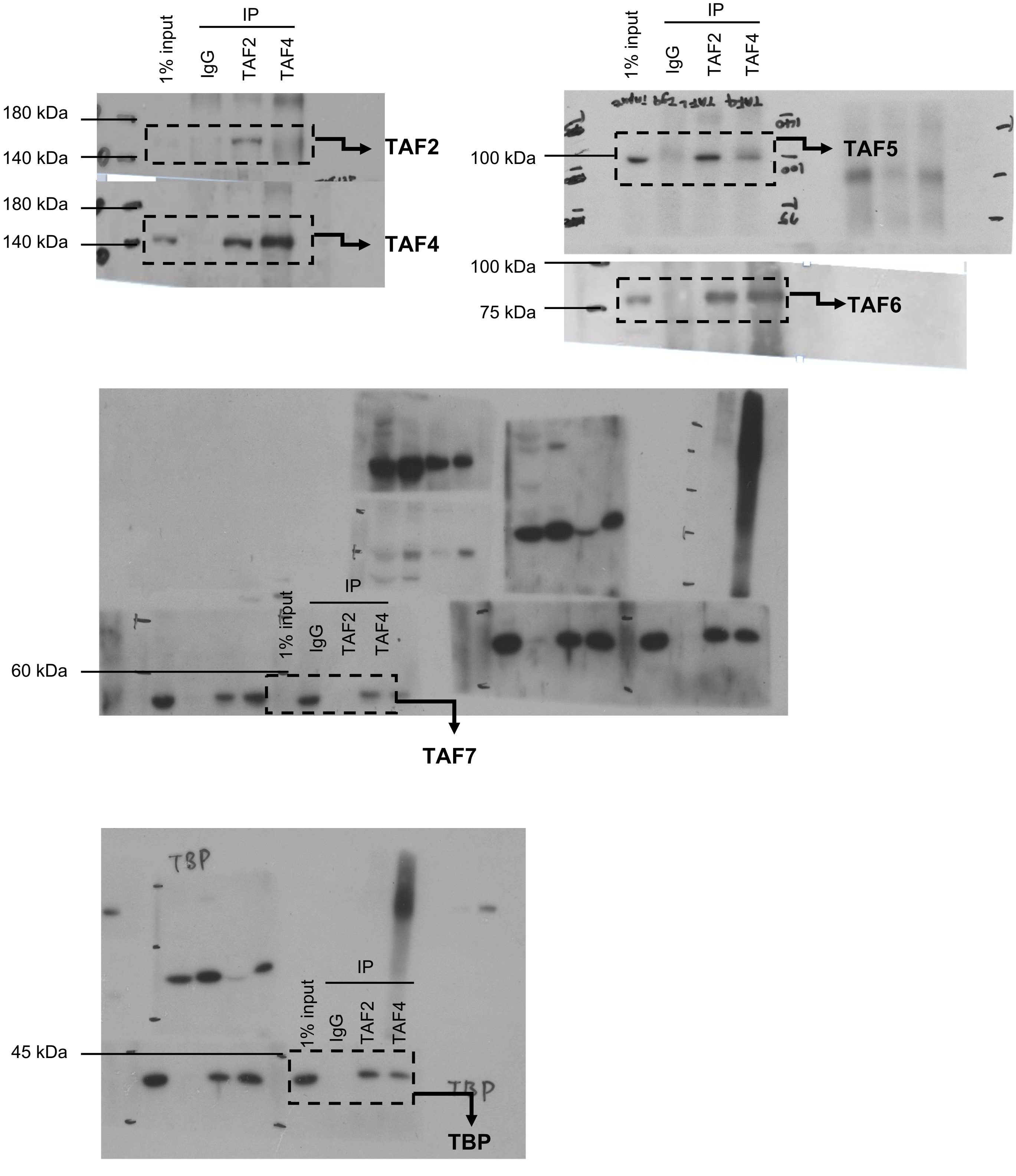

Figure 1A (A2780)

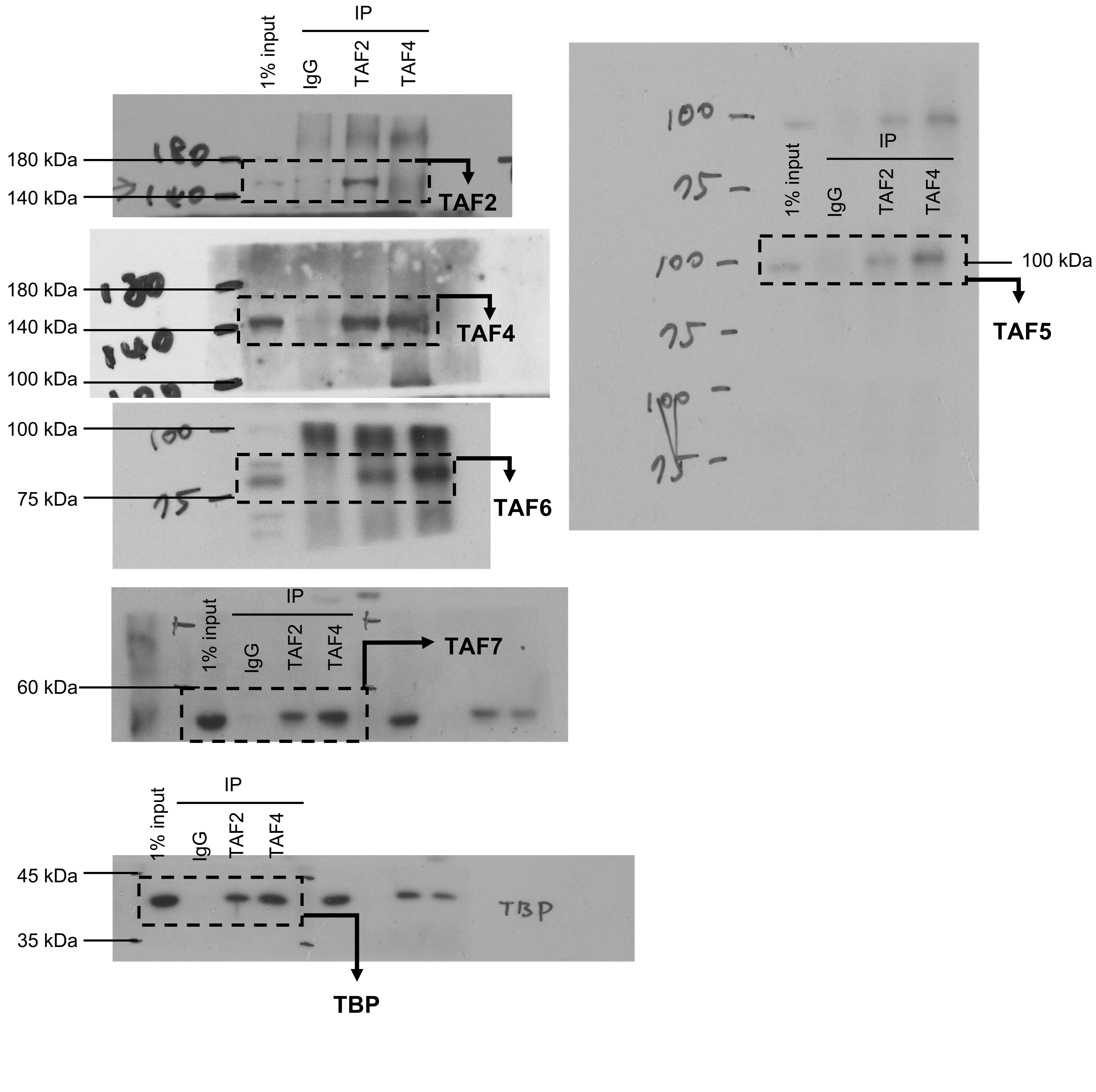

Figure 1B

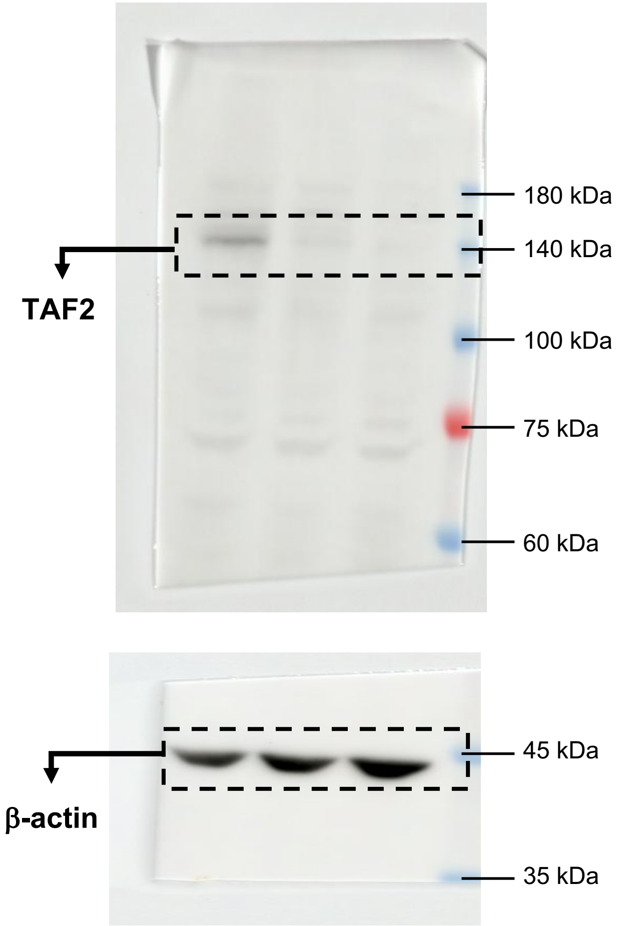

Figure 1C

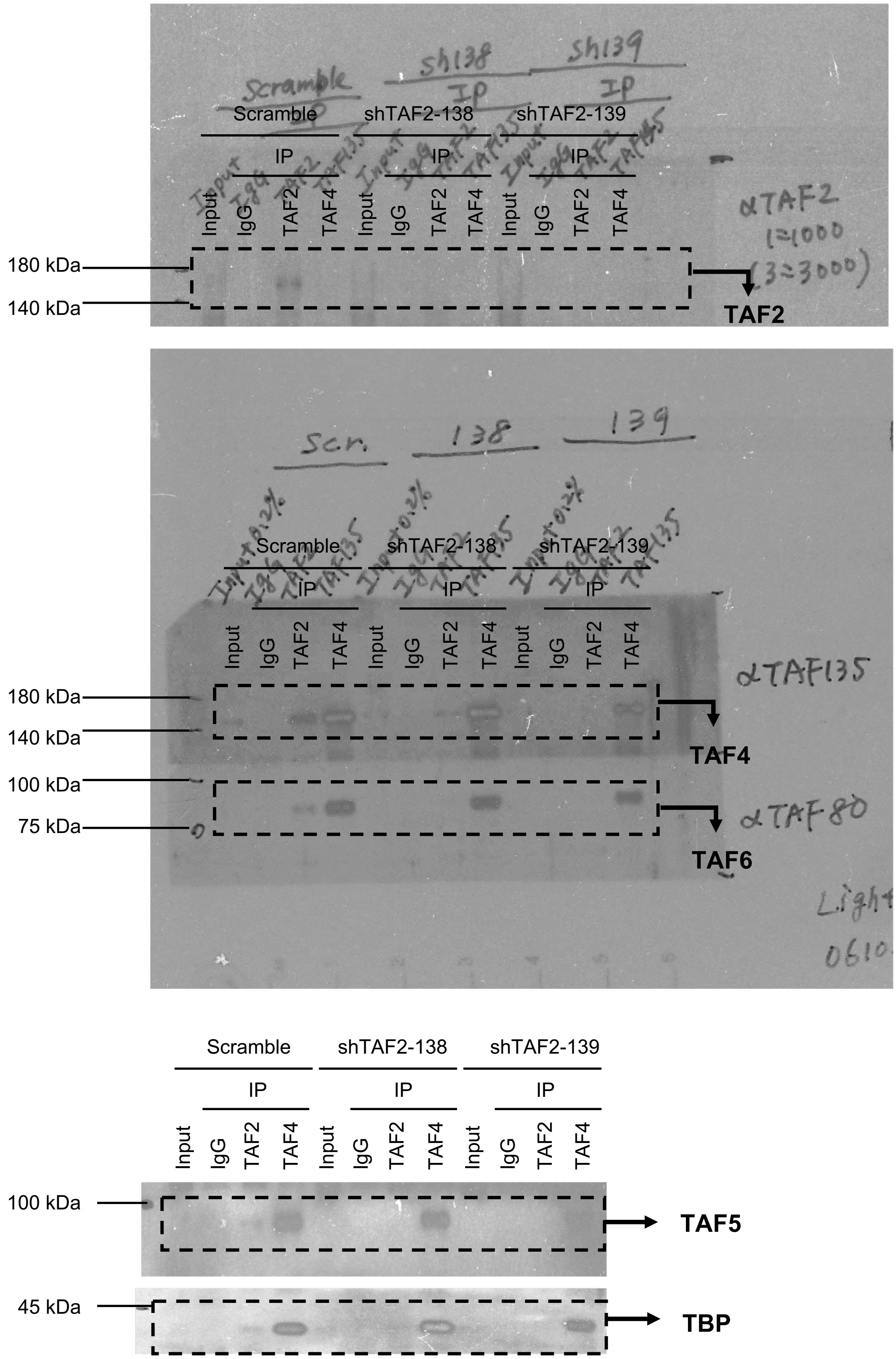
Scramble
shTAF2-138
shTAF2-139
IP
Input
IgG
TAF2
TAF4
180 kDa
140 kDa
TAF2
TAF4
100 kDa
75 kDa
TAF6
TAF5
45 kDa
TBP

Figure 4B

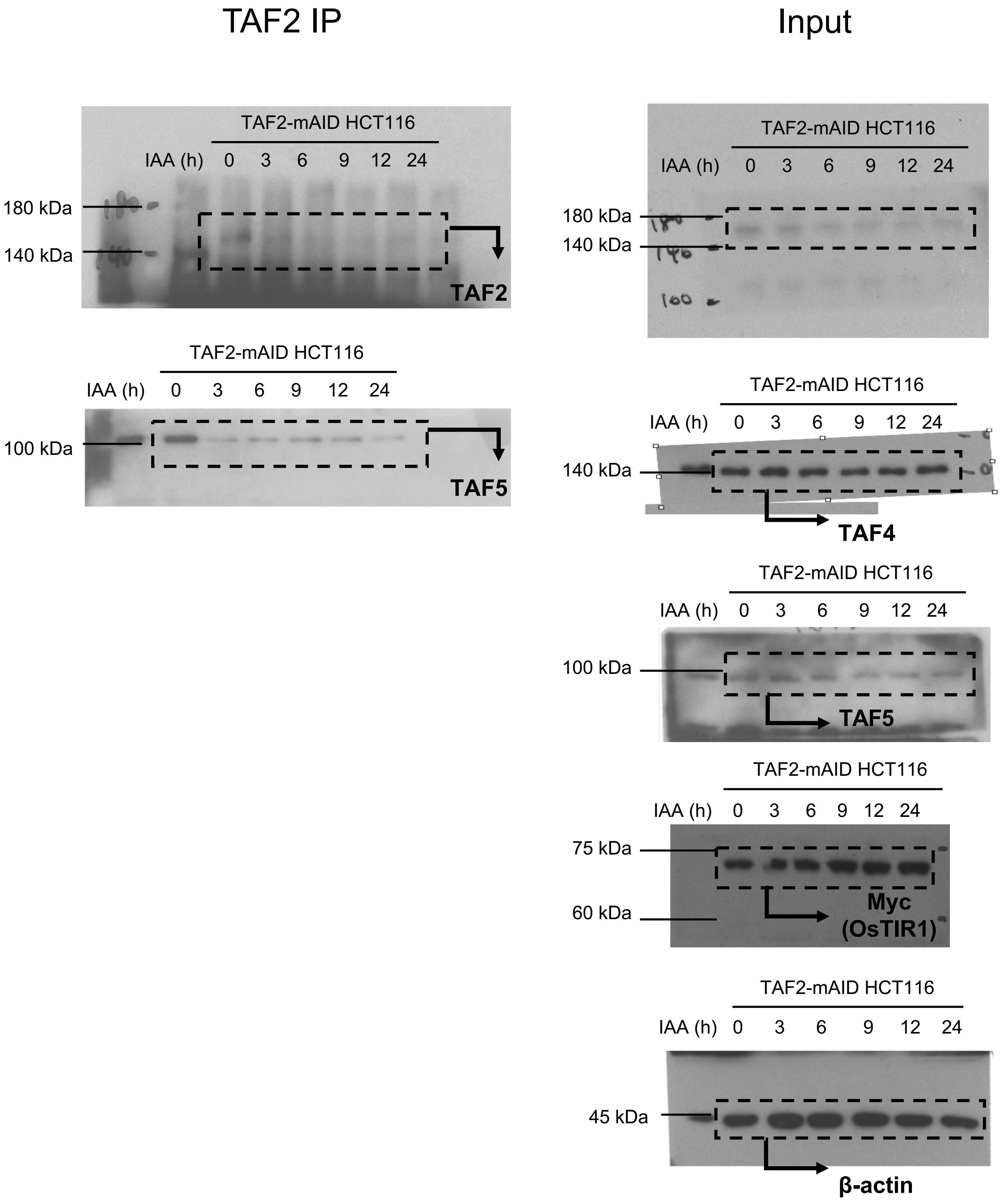

TAF2 IP
Input
TAF2-mAID HCT116
IAA (h) 0 3 6 9 12 24
180 kDa
140 kDa
TAF2
100 kDa
TAF5
TAF4
75 kDa
60 kDa
Myc (OsTIR1)
45 kDa
β-actin

Figure 6F

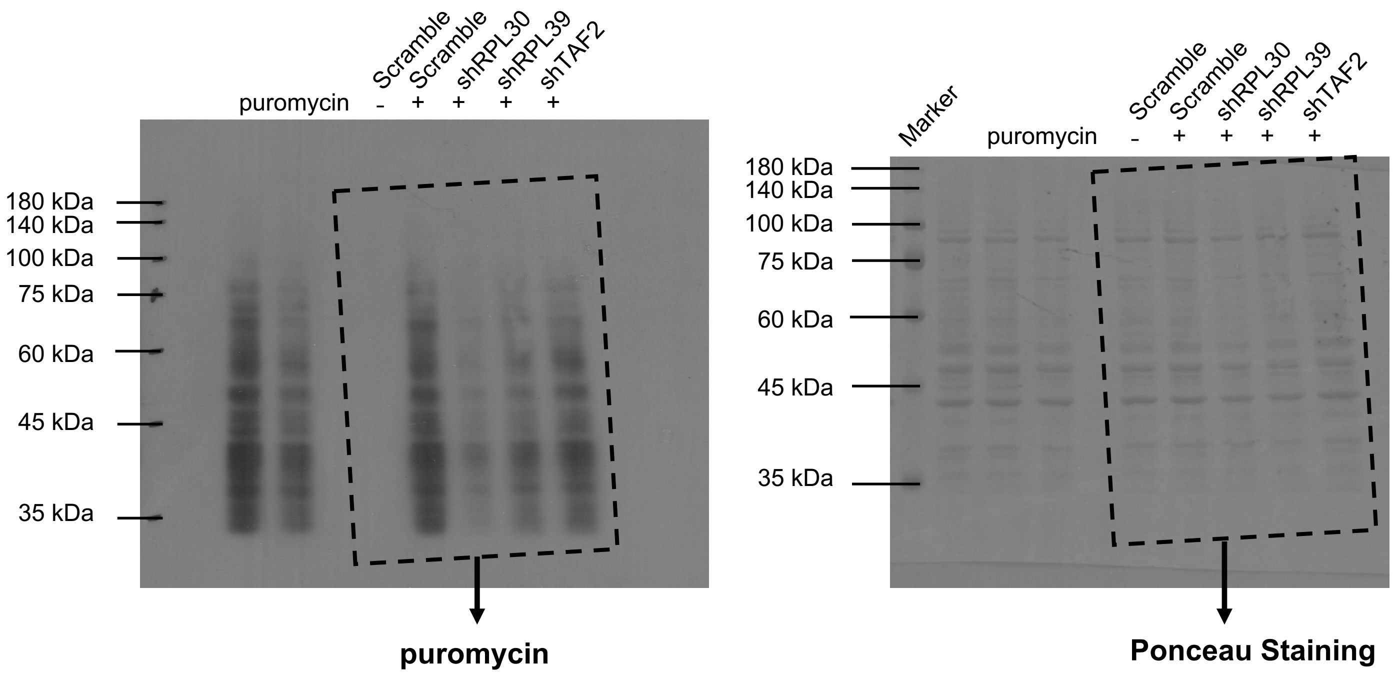

Scramble
Scramble
shRPL30
shRPL39
shTAF2
puromycin - + + + +
180 kDa
140 kDa
100 kDa
75 kDa
60 kDa
45 kDa
35 kDa
puromycin
Marker
Scramble
Scramble
shRPL30
shRPL39
shTAF2
puromycin - + + + +
180 kDa
140 kDa
100 kDa
75 kDa
60 kDa
45 kDa
35 kDa
Ponceau Staining

Supplementary Figure S1

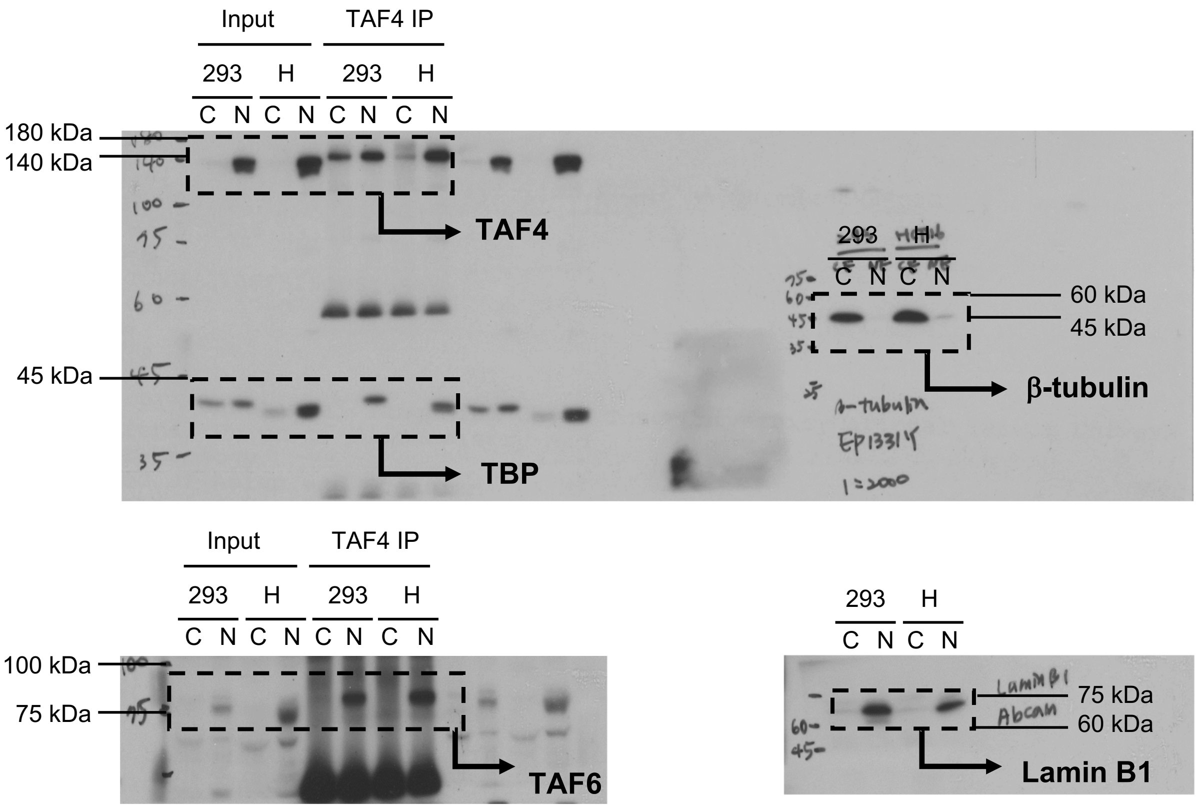

Supplemental Figure S5

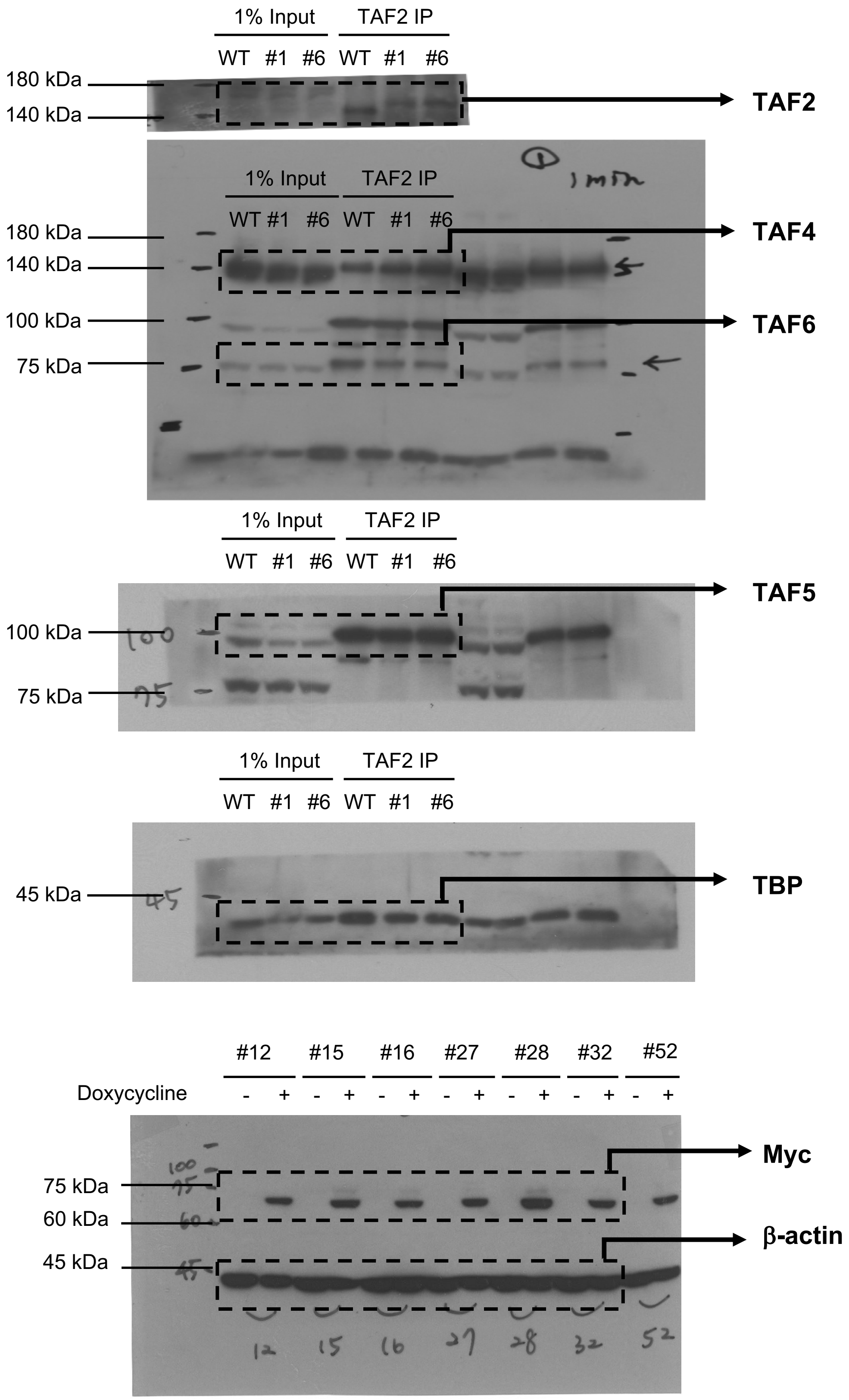

Supplement: Supplementary file 2 — Original Immunoblot Images [file 41420_2024_2017_MOESM2_ESM.pdf]
